# One-Pot Synthesis of Novel Chiral *β*-Amino Acid Derivatives by Enantioselective Mannich Reactions Catalyzed by Squaramide Cinchona Alkaloids

**DOI:** 10.3390/molecules18066142

**Published:** 2013-05-23

**Authors:** Kankan Zhang, Xueping Liang, Ming He, Jian Wu, Yuping Zhang, Wei Xue, Linhong Jin, Song Yang, Deyu Hu

**Affiliations:** State Key Laboratory of Breeding Base of Green Pesticide and Agricultural Bioengineering, Key Laboratory of Green Pesticide and Agricultural Bioengineering, Ministry of Education, Guizhou University, Guiyang 550025, China; E-Mails: kankan16@126.com (K.Z.); lxpxueping@126.com (X.L.); hmher@126.com (M.H.); jianwu2691@yahoo.com.cn (J.W.); zhangyupinggz@163.com (Y.Z.); shouldww@126.com (W.X.); fcc.jinlh@gzu.edu.cn (L.J.); fcc.syang@gzu.edu.cn (S.Y.)

**Keywords:** Asymmetric Mannich addition, *β*-amino acid derivatives, 1,3,4-thiadiazole moiety, thiourea derived from squaramide cinchona alkaloid

## Abstract

An efficient one-pot synthesis of novel *β*-amino acid derivatives containing a thiadiazole moiety was developed using a chiral squaramide cinchona alkaloid as organocatalyst. The reactions afforded chiral *β*-amino acid derivatives in moderate yields and with moderate to excellent enantioselectivities. The present study demonstrated for the first time the use of a Mannich reaction catalyzed by a chiral bifunctional organocatalyst for the one-pot synthesis of novel *β*-amino acid derivatives bearing a 1,3,4-thiadiazole moiety on nitrogen.

## 1. Introduction

*β*-Amino acids are key structural components of peptides, peptidomimetics, and natural products [1,2]. Stereochemically defined *β*-amino acids are synthesized and applied in drug development, molecular recognition, and bimolecular structure and functional studies [3,4,5,6,7]. Extensive research has been carried out to develop suitable methodology for the stereoselective synthesis of *β*-amino acids [8,9,10,11,12,13,14]. The general methods usually rely on classical resolution, stoichiometric use of chiral auxiliaries, or homologation of *α*-amino acids [15,16,17,18,19,20,21]. However, the synthesis of *β*-amino acids bearing various functional groups on the *β*-carbon, while maintaining desired chirality, is a big challenge. Thiadiazole ring systems have been well studied and reported to have a variety of biological activities, including antifungal, antitubercular, antibacterial, anticancer, and analgesic properties [22,23,24,25,26]. Therefore, enantio-enriched *β-*amino acids containing 1,3,4-thiadiazoles have potential therapeutic value and are a great challenge for chiral synthesis.

Our group has previously developed a highly enantioselective Mannich reaction catalyzed by cinchona alkaloid thiourea to produce novel *β*-amino acid ester and ketone derivatives containing benzoxazol and benzothiazole moieties, and *β*-amino ketones containing benzothiazole units [27,28,29]. In the present work, we report an enantioselective synthesis of *β*-amino acid derivatives bearing a 1, 3, 4-thiadiazole moiety on nitrogen by an asymmetric Mannich reaction catalyzed by a squaramide cinchona alkaloid catalyst. To the best of our knowledge, our study is the first one on the asymmetric synthesis of *β*-amino acid derivatives containing a 1,3,4-thiadiazole moiety on nitrogen in the presence of a squaramide organocatalyst.

## 2. Results and Discussion

### 2.1. Chemistry

**Scheme 1:**
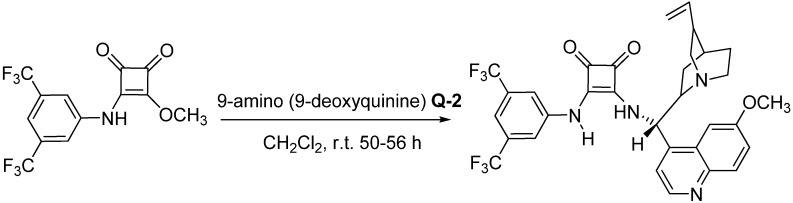
Synthesis of catalyst **SQ**.

In prior studies, several differentially substituted chiral derivatives of squaramides (SQ) have been synthesized and used as catalysts for asymmetric reactions [30,31,32,33,34]. The preparation of SQ involves an easy one-step synthesis as shown in Scheme 1. 3-(3,5-Bis(trifluoromethyl)phenylamino)-4-methoxycyclobut-3-ene-1,2-dione was stirred with 9-amino (9-deoxyquinine) in dry DCM at room temperature to produce the catalyst SQ in moderate yield. 

**Figure 1:**
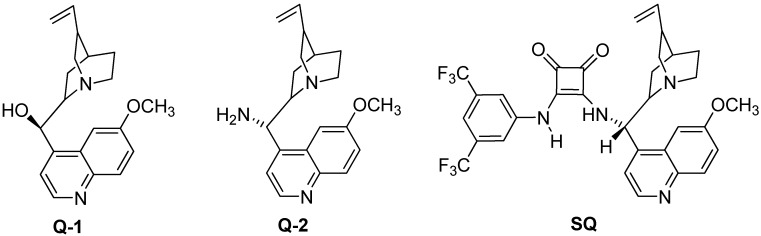
Structures of commercial cinchona alkaloid catalysts.

**Scheme 2:**
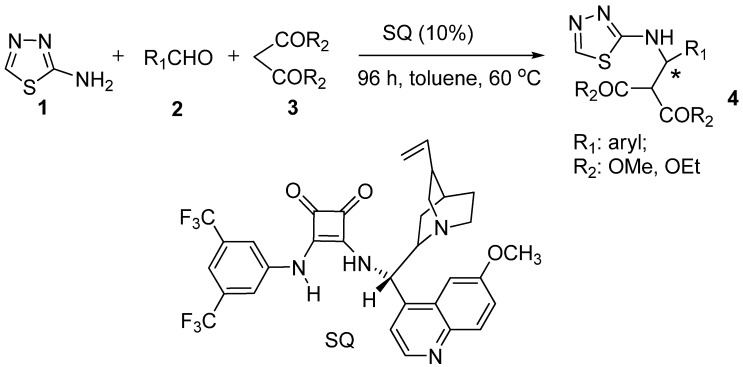
Synthesis of the chiral *β*-amino acid derivatives.

In search of the optimum catalyst for the enantioselective synthesis of *β-*amino acids containing 1,3,4-thiadiazoles, we initially tested two commercially available cinchona alkaloid catalysts **Q-1** and **Q-2** (Figure 1) in the catalytic one-pot asymmetric Mannich reaction of 2-amino-1,3,4-thiadiazole (**1**), benzaldehyde (**2**, R_1_ = Ph) and dimethyl malonate (**3**, R_2_ = Me) (Scheme 2). 

The quinine catalyst **Q-1** turned out to be a poor catalyst, affording a 37% yield and 11% *ee*, whereas the catalyst **Q-2** performed slightly better, with 39% yield and 37% *ee*, respectively, when the reactions were carried out at 60 °C for 96 h (entry 1 and 2, Table 1). Since both **Q-1** and **Q-2** failed to achieve the desired high yield and enantioselectivity, we continued our search for a better catalyst and found that the cinchona alkaloid derivative (**SQ**) bearing both the hydrogen-bond donor squaramide and the hydrogen-bond acceptor tertiary amines delivered superior results. Compared with the quinine **Q-1** and 9-amino (9-deoxyquinine) **Q-2** catalysts, the **SQ** catalyst bearing strong electron-withdrawing trifluoromethyl substituents on its benzene ring achieved better yield (41%) and much higher enantioselectivity (91%) (entry 3, Table 1). The superior performance of SQ catalyst could be attributed to its ability to promote the reaction through double-hydrogen bond activation of the substrate.

**Table 1:** Screening of various catalysts ^a^.

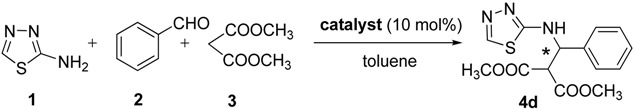					
Entry	Catalyst	Temp. (°C)	Solvent	Yield ^b^ (%)	*ee* ^c^ (%)
1	**Q-1**	60	Toluene	37	11
2	**Q-2**	60	Toluene	39	37
3	**SQ**	60	Toluene	41	91

^a^ Reactions were carried out with 1.0 mmol of **1**, 1.2 mmol of **2**, and 1.5 mmol of **3** in 3.0 mL of toluene in the presence of 10 mol% catalyst at 60 °C for 96 h. ^b^ Isolated yield after chromatographic purification. ^c^ ee Determined by HPLC analysis (Chiralpak IA).

The effects of three important experimental parameters (solvent, catalyst loading, and reaction temperature) on the **SQ-**catalyzed reactions were examined to determine the optimal reaction conditions (Table 2). We found that solvent significantly affected the reaction yield and *ee* of the final product. Among the four solvents tested, the best yield was obtained in methanol (53%) while the highest *ee* was achieved in toluene (91%). (entries 1–4, Table 2). The catalyst loading also affected the yield and *ee* of **4d**. When the reaction was conducted at 60 °C in toluene, the highest yield (49%; entry 4, Table 2) and *ee* (91%; entry 4, Table 2) were obtained with the highest catalyst loading of 10 mol%. The yield and *ee* were also affected by the reaction temperature. When the reaction temperature was increased from room temperature to 60 °C, the reaction yield and *ee* increased by 12% and 11%, respectively (entries 4 and 5, Table 2). Taken together, the optimum result was achieved at 60 °C with 10 mol% catalyst loading in toluene.

**Table 2:** Optimization of reaction conditions using catalyst **SQ** ^a^.

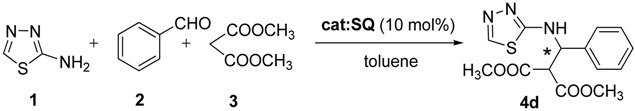					
Entry	Catalyst load (mol%)	Temp. (°C)	Solvent	Yield ^b^ (%)	*ee* ^c^ (%)
1	10	60	Methanol	53	71
2	10	60	Acetone	41	75
3	10	60	Chloroform	38	83
4	10	60	Toluene	49	91
5	10	r.t.	Toluene	37	80
6	5	60	Toluene	45	70
7	2.5	60	Toluene	44	43

^a^ Reactions were carried out with 1.0 mmol of **1**, 1.2 mmol of **2**, and 1.5 mmol of **3 **in 2.0 mL of the specified solvent for 96 h. ^b^ Isolated yield after chromatographic purification. ^c^ ee Determined by HPLC analysis (Chiralpak IA).

Having established the ideal reaction conditions, we explored the synthetic scope of the reaction with different aldehydes and malonates as substrates. The results were summarized in Table 3. The highest enantioselectivities were obtained with a phenyl R_1_ group with higher than 45% yields (45% yield, 91% *ee*, entry 5; 52% yield, 99% *ee*, entry 6, Table 3). However, when the R_1_ group was substituted phenyl, the yields dropped below 45% (except product **4e**, 61% yield, entry 4, Table 3) and the *ee* values were < 60%, regardless of the substituent being an electron-withdrawing group (chlorine and trifluoromethyl) or an electron-donating group (methoxyl) (entries 1–4, Table 3). Therefore, the reaction showed highest enantioselectivity and best chemical yields with unsubstituted benzaldehyde.

**Table 3:** Enantioselective Mannich reaction of 2-amino-1, 3, 4-thiadiazole, aldehyde, and malonate catalyzed by the **SQ** catalyst ^a^.

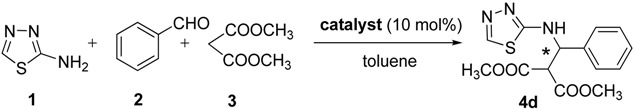						
Entry	4	R_1_	R_2_	Time (h)	Yield ^b^ (%)	*ee* ^c^ (%)
1	**4a**	2, 3-di-Cl-Ph-	OMe	96	42	41
2	**4b**	3-CF_3_- Ph-	OMe	96	39	58
3	**4c**	2, 3-di-OMe-Ph-	OMe	97	44	48
4	**4d**	Ph-	OMe	96	45	91
5	**4e**	2, 4-di-Cl-Ph-	OMe	96	61	42
6	**4f**	Ph-	OEt	93	52	99

^a^ Reactions were carried out with 1.0 mmol of **1**, 1.2 mmol of **2**, and 1.5 mmol of **3** in 2.0 mL of toluene in the presence of 10 mol% catalyst **SQ** at 60 °C for 90–110 h. ^b^ Isolated yields after chromatographic purification. ^c^ ee Determined by HPLC analysis (Chiralpak IA).

2-Amino-1,3,4-thiadiazole (**1**) aldehyde **2**, and dimethyl malonate (or diethyl malonate) **3** were mixed in the presence of catalyst **SQ** and stirred at 60 °C for 93–97 h. The *in situ* generation of imine was confirmed by thin-layer chromatography (TLC) and mass spectrometry (MS). 

**Scheme 3:**
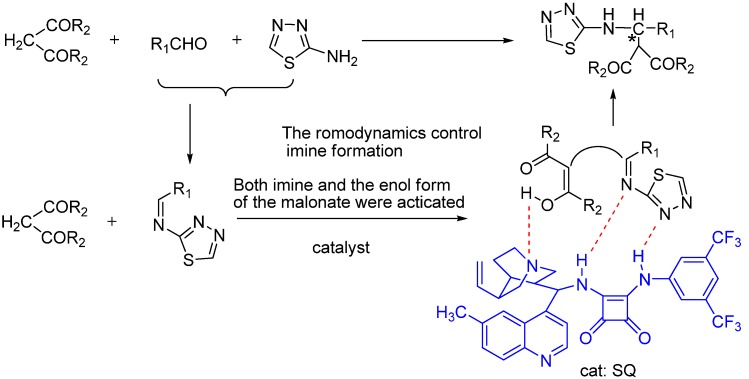
Proposed mechanism of the asymmetric Mannich reaction catalyzed by squaramide cinchona alkaloid.

We speculate that while the imine was activated by the squaramide moiety through hydrogen bonding, the intermediate transition state (enol form of the malonate) was activated by the basic nitrogen atom in the tertiary amine moiety of the catalyst, leading to a stable transition state (Scheme 3). These speculated interactions in our proposed mechanism might be responsible for the observed stereochemical outcome of the reaction and the enhanced reaction rate.

## 3. Experimental

### 3.1. General

Unless otherwise stated, all reagents and reactants were purchased from commercial suppliers. Melting points were determined with a XT-4 binocular microscope (Beijing Tech Instrument Co., Beijing, China) without correction. ^1^H-NMR, ^13^C-NMR, and ^1^^9^F-NMR spectra were recorded on a JEOL ECX 500 NMR spectrometer at room temperature operated at 500 MHz for ^1^H-NMR, 125 MHz for ^13^C-NMR, and 470 MHz for ^1^^9^F-NMR using CDCl_3_ as solvent and TMS as an internal standard. Infrared spectra were recorded in KBr on a Bruker VECTOR 22 spectrometer, and elemental analysis was performed on an Elemental Vario-III CHN analyzer. The progress of the reactions was monitored by TLC, and preparative TLC was performed on silica gel GF_254_. High-performance liquid chromatography (HPLC) analysis was performed on an Agilent 1100/1200 series instrument equipped with a quaternary pump using a Daicel Chiralpak IA Column (250 mm × 4.6 mm). UV absorption was monitored at 270 nm. Specific rotations were measured on a WZZ-2S digital polarimeter with a sodium lamp. The intermediate 3-(3,5-bis(trifluoromethyl)phenylamino)-4-methoxycyclobut-3-ene-1,2-dione was prepared according to a literature procedure [26].

### 3.2. Preparation of the ***SQ*** Catalyst (Scheme 1)

A solution of 3-(3,5-bis(trifluoromethyl)phenylamino)-4-methoxycyclobut-3-ene-1,2-dione (0.339 g, 1.0 mmol) in dry DCM (5.0 mL) was added dropwise at room temperature to a solution of 9-amino- (9-deoxyquinine) (0.330 g, 1.02 mmol) in dry DCM (5.0 mL). The reaction mixture was stirred at room temperature for 50–56 h. The solvent was removed *in vacuo* and the residue was purified by column chromatography (silica gel: ethyl acetate/methanol = 15/1) to generate an amorphous solid (59% yield) with a m.p. of 170–171 °C; [α]_D_^2^^5^ +56.8 (c = 0.50, DMSO); ^1^H-NMR (CDCl_3_): *δ* 8.62 (s, 1H), 8.00 (d, *J* = 5.0 Hz, 1H), 7.70–7.69 (m, 1H), 1.60–7.54 (m, 2H), 7.39 (s, 2H), 7.36 (s, 1H), 6.12 (br s, 1H), 5.77–5.70 (m, 1H), 5.02 (br s, 1H), 4.98 (d, *J* = 10.0 Hz, 1H), 3.93 (s, 3H), 3.38–3.34 (m, 2H), 3.14–3.12 (m, 1H), 2.80–2.75 (m, 2H), 2.33 (br s, 1H), 2.05 (s, 1H), 1.73 (s, 1H), 1.65 (s, 2H), 0.87 (br s, 1H); ^13^C-NMR (CDCl_3_): *δ* 184.7, 180.7, 168.9, 163.6, 159.3, 147.1, 144.2, 141.0, 140.8, 132.5, 130.4, 128.1, 124.3, 132.2, 122.1, 118.1, 115.4, 114.1, 100.7, 59.8, 55.6, 53.5, 40.5, 39.2, 27.5, 26.9, 25.9; ^19^F-NMR (CDCl_3_, ppm): *δ* -63.09; IR (KBr, cm^−1^): *ν* 3212, 3087, 2945, 2877, 1794, 1691, 1623, 1606, 1554, 1509, 1471, 1436, 1380, 1179, 1230, 1181, 1133, 1029, 934, 881, 850, 830, 700, 680*.* MASS (ESI) m/z calcd. for C_32_H_29_F_6_N_4_O_3_ [M+H]^+^ 631.3, found 631.3.

### 3.3. Preparation of Chiral Compounds **4a**–**4f**

Aldehydes **2 **(0.60 mmol) and chiral catalyst **SQ** (0.005 mmol) were added to a well-stirred solution of 2-amino-1,3,4-thiadiazole (**1**, 0.50 mmol), and dimethyl malonate (diethyl malonate) **3** (0.75 mmol) in toluene (2.5 mL). The mixtures were stirred at 60 °C for 96 h and reaction progress monitored by TLC. After the reactions were completed, the solvents were evaporated and the crude products were directly purified by preparative TLC (GF254 silica gel) using a mixture of petroleum ether and ethyl acetate (1/1–2/3, v/v) as developing solvent to produce chiral compounds **4a**-**4f**.

*Dimethyl 2-((1,3,4-thiadiazol-2-ylamino)(2,3-dichlorophenyl)methyl)malonate* [**(-)-4a**]: White solid, yield 42%; m.p. 127–128 °C; *ee* 41% as determined by HPLC [Daicel Chiralpak IA, hexane/EtOH = 80/20, 1.0 mL·min^−1^, *λ* = 270 nm, tr (major) = 10.83 min, tr (minor) = 13.29 min], [α]_D_^2^^0^ −30.2 (*c* 1.01, CHCl_3_); ^1^H-NMR (CDCl_3_): *δ* 8.40 (s, 1H, NH), 7.61 (s, 1H, ArH), 7.19–7.16 (m, 2H, ArH), 5.89 (s, 1H, =CH), 4.19 (d, *J* = 4.6 Hz, 1H, CH), 3.77 (s, 3H, OCH_3_), 3.66 (s, 3H, OCH_3_); ^13^C-NMR (CDCl_3_): *δ* 168.4, 168.2, 166.9, 142.7, 137.6, 133.7, 131.3, 130.5, 127.7, 126.3, 57.5, 53.9, 53.4, 52.9; IR (KBr) *ν*: 3401, 3223, 3024, 2953, 2848, 1749, 1734, 1558, 1506, 1498, 1273, 1247, 1157, 1031, 1039, 893, 788, 752 cm^−1^; Anal. Calcd for C_14_H_1__3_Cl_2_N_3_O_4_S: C 43.09, H 3.36, N 10.77; found: C 43.26, H 3.18, N 10.64.

*Dimethyl* *2-((1,3,4-thiadiazol-2-ylamino)(3-(trifluoromethyl)phenyl)methyl)* *malonate* [**(-)-4b**]: White solid, yield 39%; m.p. 156–157 °C; *ee* 58% as determined by HPLC [Daicel Chiralpak IA, hexane/EtOH = 80/20, 1.0 mL·min^−1^, *λ* = 270 nm, tr (major) = 7.75 min, tr (minor) = 10.46 min], [α]_D_^2^^5^ −73.4 (*c* 0.57, CHCl_3_); ^1^H-NMR (CDCl_3_): *δ* 8.40 (s, 1H, NH), 7.62–7.59 (m, 2H, ArH), 7.56–7.55(m, 1H, ArH), 7.49–7.47 (m, 1H, ArH), 5.66 (d, *J* = 5.0 Hz, 1H, =CH), 4.00 (s, 1H, CH), 3.73 (s, 3H, OCH_3_), 3.69 (s, 3H, OCH_3_); ^13^C-NMR (CDCl_3_): *δ* 168.3, 168.1, 166.9, 142.6, 139.3, 131.4, 131.1, 130.3, 129.5, 125.3, 123.5, 58.9, 56.7, 53.3, 53.1; ^19^F-NMR (CDCl_3_): *δ* -62.7; IR (KBr): *ν* 3445, 3225, 3024, 2920, 2851, 1747, 1722, 1558, 1506, 1458, 1437, 1329, 1265, 1163, 1072, 1024, 893, 704 cm^−1^; Anal. Calcd for C_15_H_14_F_3_N_3_O_4_S: C 46.27, H 3.62, N 10.79; found: C 46.59, H 3.42, N 10.54.

*Dimethyl 2-((1,3,4-thiadiazol-2-ylamino)(2,3-dimethoxyphenyl)methyl) malonate* [**(-)-4c**]: White solid, yield 44%; m.p. 102–103 °C; *ee* 48% as determined by HPLC [Daicel Chiralpak IA, hexane/EtOH = 80/20, 1.0 mL·min^−1^, *λ* = 270 nm, tr (major) = 10.83 min, tr (minor) = 12.43 min], [α]_D_^2^^0^ −22.4 (*c* 0.65, CHCl_3_); ^1^H-NMR (CDCl_3_): *δ* 8.37 (s, 1H, NH), 6.98–6.97 (m, 1H, ArH), 6.96–6.95 (m, 1H, ArH), 6.86–6.85 (m, 1H, ArH), 5.62 (d, *J* = 5.0 Hz, 1H, =CH), 4.14 (d, *J* = 5.0 Hz, 1H, CH), 4.03 (s, 3H, OCH_3_), 3.86 (s, 3H, OCH_3_), 3.71 (s, 3H, CH_3_), 3.65 (s, 3H, CH_3_); ^13^C-NMR (CDCl_3_): *δ* 169.0, 168.6, 167.3, 152.6, 146.5, 142.3, 130.8, 124.0, 119.5, 112.7, 60.9, 56.7, 55.8, 55.7, 53.1, 52.8; IR (KBr): *ν* 3443, 3186, 3107, 2943, 2837, 1761, 1734, 1558, 1481, 1437, 1364, 1271, 1223, 1063, 1001, 812, 794, 743 cm^−1^; Anal. Calcd for C_16_H_19_N_3_O_6_S: C 50.39, H 5.02, N 11.02; found: C 50.60, H 5.09, N 11.16.

*Dimethyl 2-((1,3,4-thiadiazol-2-ylamino)(phenyl)methyl)malonate* [**(-)-4d**]: White solid, yield 45%; m.p. 162–163 °C; *ee* 91% as determined by HPLC [Daicel Chiralpak IA, hexane/EtOH = 80/20, 1.0 mL·min^−1^, *λ* = 270 nm, tr (major) = 9.12 min, tr (minor) = 11.53 min], [α]_D_^20^ −75.2 (c = 1.00, CHCl_3_); ^1^H-NMR (CDCl_3_): *δ* 8.36 (s, 1H, NH), 7.39–7.38 (d, *J* = 5.0 Hz, 2H, ArH), 7.35–7.32 (m, 2H, ArH), 7.30–7.28 (m, 1H, ArH), 5.39 (d, *J* = 6.3 Hz, 1H, =CH), 4.03 (d, *J* = 6.3 Hz, 1H, CH), 3.69 (s, 3H, OCH_3_), 3.67 (s, 3H, OCH_3_); ^13^C-NMR (CDCl_3_): *δ* 169.5, 168.2, 167.0, 142.1, 137.6, 130.1, 128.9, 128.5, 128.4, 126.9, 60.5, 57.4, 53.1, 53.0; IR (KBr): *ν* 3221, 3011, 2953, 1734, 1558, 1506, 1435, 1259, 1238, 1060, 1022, 891, 769, 704 cm^−1^; Anal. Calcd for C_14_H_15_N_3_O_4_S: C 52.33, H 4.70, N 13.08; found: C 52.44, H 4.63, N 13.10.

*Diethyl 2-((1,3,4-thiadiazol-2-ylamino)(2,4-dichlorophenyl)methyl)malonate* [**(-)-4e**]: White solid, yield 61%; m.p. 142–143 °C; *ee* 42% as determined by HPLC [Daicel Chiralpak IA, hexane/EtOH = 80/20, 1.0 mL·min^−1^, *λ* = 270 nm, tr (major) = 8.87 min, tr (minor) = 13.87 min], [α]_D_^20^ −151.3 (c = 0.84, CHCl_3_); ^1^H-NMR (CDCl_3_): *δ* 8.39 (s, 1H, NH), 7.42–7.41 (m, 1H, ArH), 7.40–7.38 (m, 1H, ArH), 7.22–7.21 (m, 1H, ArH), 5.80 (d, *J* = 4.6 Hz, 1H, =CH), 4.23–4.18 (m, 2H, OCH_2_), 4.11–4.08 (m, 2H, OCH_2_), 4.06 (d, *J* = 6.3 Hz, 1H, CH), 1.22 (t, *J* = 6.9 Hz, 3H, CH_3_), 1.17 (t, *J* = 7.1 Hz, 3H, CH_3_); ^13^C-NMR (CDCl_3_): *δ* 168.3, 168.0, 166.5, 142.6, 134.8, 134.0, 133.8, 129.8, 129.4, 127.7, 62.5, 62.2, 56.8, 54.4, 14.0, 13.9; IR (KBr): *ν* 3362, 3109, 3088, 2990, 2938, 1738, 1719, 1585, 1558, 1497, 1472, 1298, 1240, 1171, 1099, 1059, 866, 781, 743 cm^−1^; Anal. Calcd for C_16_H_17_Cl_2_N_3_O_4_S: C 45.94, H 4.10, N 10.05; found: C 45.75, H 4.00, N 10.10.

*Diethyl 2-((1,3,4-thiadiazol-2-ylamino)(phenyl)methyl)malonate* [**(-)-4f**]: White solid, yield 52%; m.p. 120–121 °C; *ee* 99% as determined by HPLC [Daicel Chiralpak IA, hexane/EtOH = 80/20, 1.0 mL·min^−1^, *λ* = 270 nm, tr (major) = 5.61 min, tr (minor) = 9.50 min], [α]_D_^20^ −29.2 (c = 0.89, CHCl_3_); ^1^H-NMR (CDCl_3_): *δ* 8.36 (s, 1H, NH), 7.38–7.37 (m, 2H, ArH), 7.35–7.32 (m, 2H, ArH), 7.27–7.26 (m, 1H, ArH), 5.51 (s, 1H, =CH), 4.19–4.12 (m, 4H, 2OCH_2_), 3.95 (d, *J* = 5.2 Hz, 1H, CH), 1.18 (t, *J* = 7.2 Hz, 3H, CH_3_), 1.15 (t, *J* = 7.2 Hz, 3H, CH_3_); ^13^C-NMR (CDCl_3_): *δ* 169.1, 168.7, 166.6, 142.1, 137.9, 129.9, 128.4, 127.0, 62.3, 62.1, 60.3, 57.5, 14.0, 13.9; IR (KBr): *ν* 3210, 3011, 2992, 2922, 1749, 1742, 1653, 1558, 1506, 1458, 1370, 1314, 1260, 1152, 1032, 891, 876, 702 cm^−1^; Anal. Calcd for C_16_H_19_ N_3_O_4_S: C 55.00, H 5.48, N 12.03; found: C 55.11, H 5.26, N 12.05. 

## 4. Conclusions

In conclusion, we have developed a convenient one-pot synthesis of novel *β*-amino acid derivatives bearing a 1,3,4-thiadiazole moiety on nitrogen which have valuable applications in medicinal and chemical synthesis and studies using an enantioselective Mannich reaction catalyzed by the chiral squaramide cinchona alkaloid catalyst **SQ**. The desired *β*-amino acid derivatives were produced in moderate yields (39%–61%) and with moderate to excellent enantioselectivities (41%–99%). Further research aimed at investigating the mechanism and scope of the catalysts and the reactions, as well as the activity of the Mannich products against plant viruses, is underway and will be reported in due course.

## Supplementary Materials

The NMR spectra and HPLC chromatographs of catalyst **SQ** and compounds **4a**–**4f** were provided as supplementary materials can be accessed at: http://www.mdpi.com/1420-3049/18/6/6142/s1.
