# Supplementary material for: One-Pot Synthesis of Novel Chiral β-Amino Acid Derivatives by Enantioselective Mannich Reactions Catalyzed by Squaramide Cinchona Alkaloids
Source: Molecules. 2013 May 23;18(6):6142–52. doi: 10.3390/molecules18066142 (PMC6270396; doi:10.3390/molecules18066142)
Supplement: Supplementary file 1 [file molecules-18-06142-s001.pdf]

Supplementary Materials

1. ^{1}H-NMR and ^{13}C-NMR spectra of catalyst SQ and 4a–4f

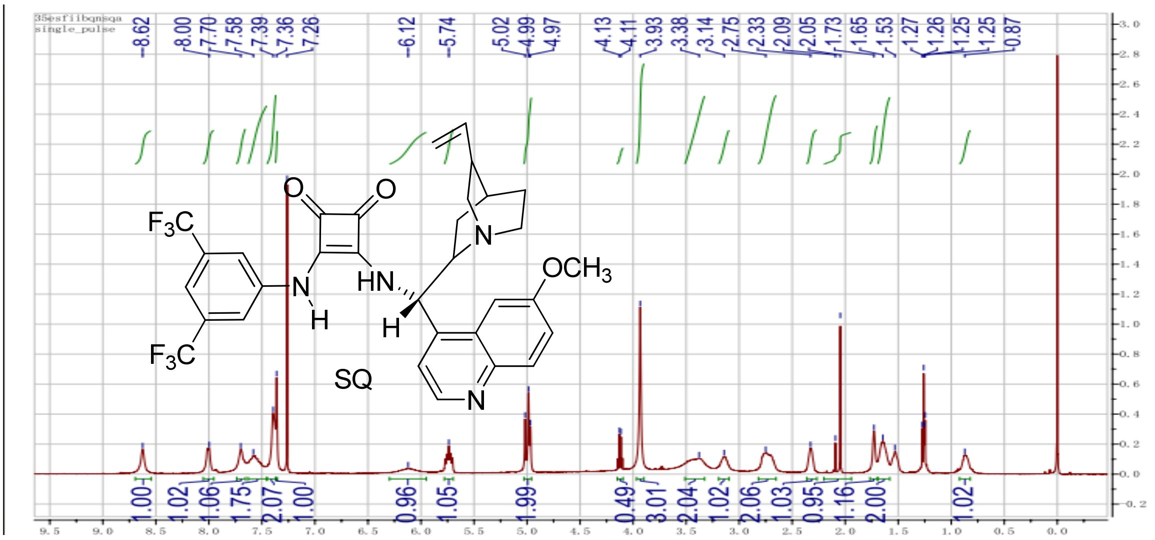

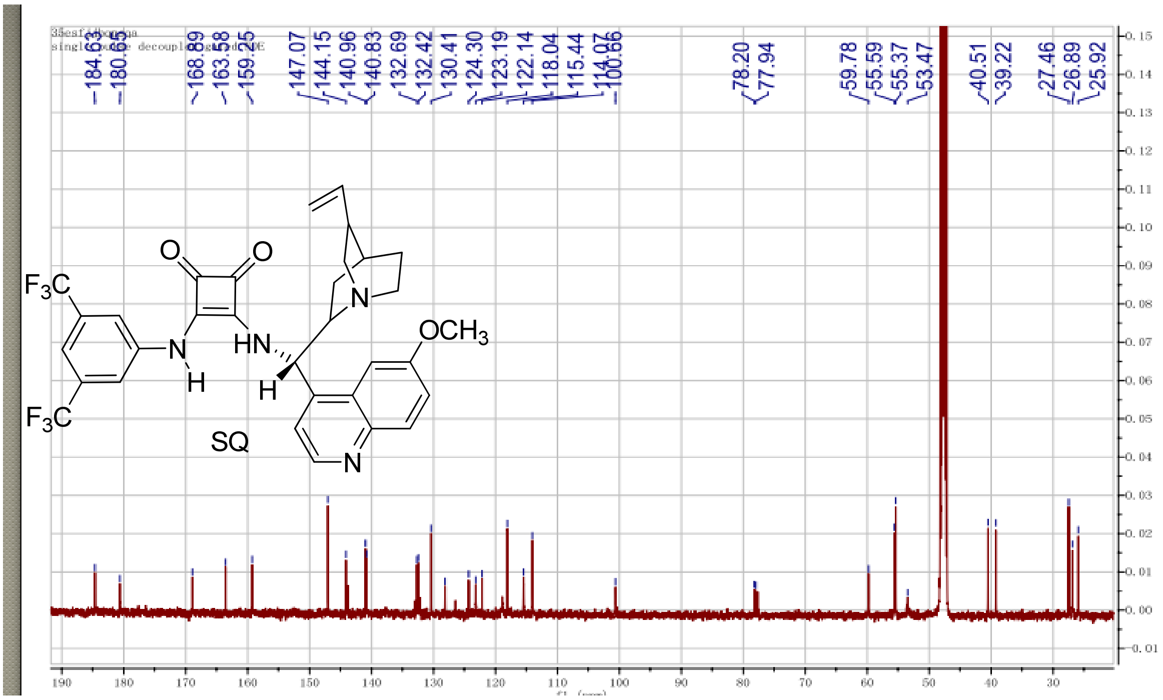

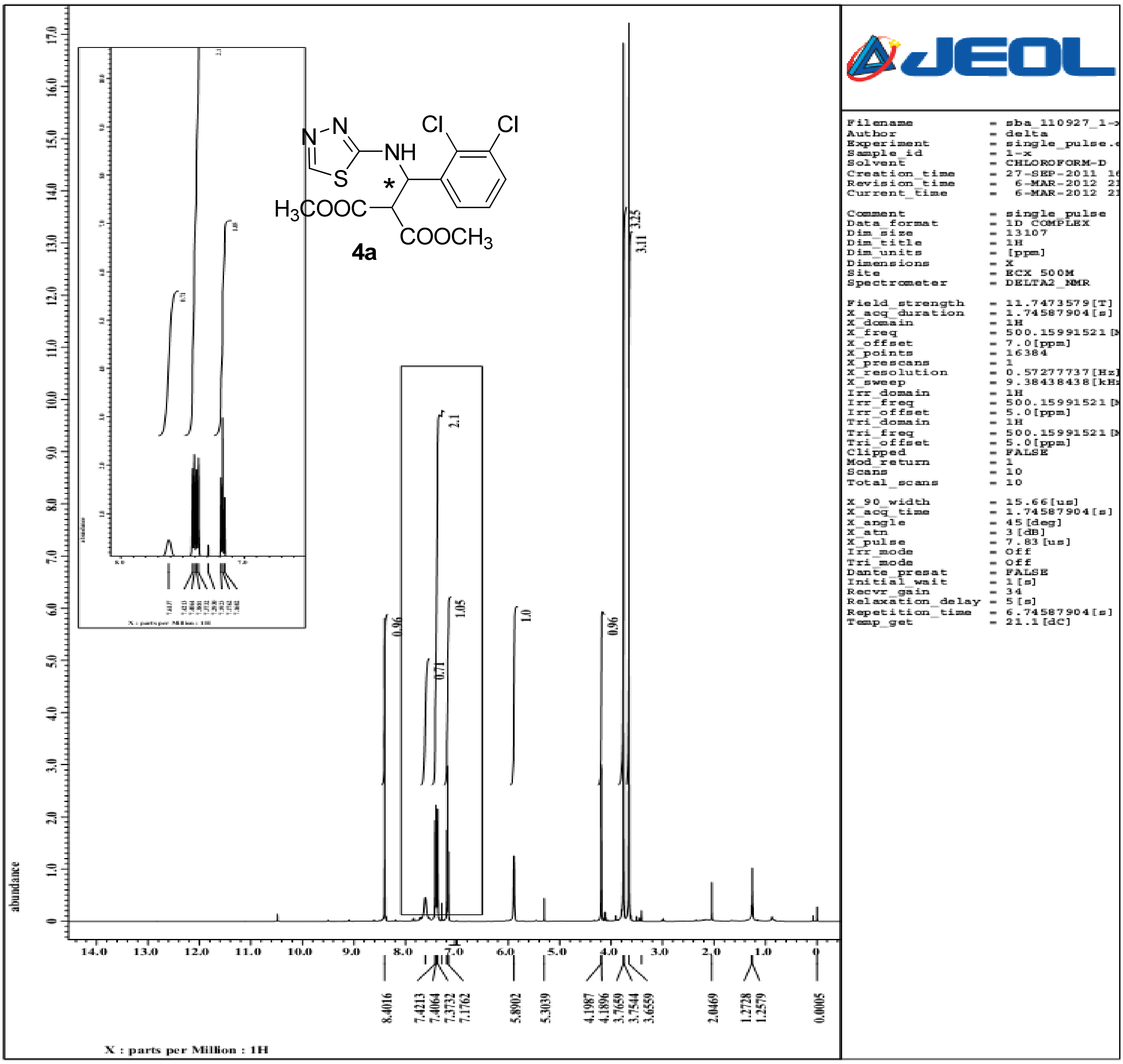
N-N
S
NH
Cl
Cl
*
H3COOC
COOCH3
4a
0.96
0.71
2.1
1.05
1.0
0.96
3.11
3.25
abundance
X : parts per Million : 1H
8.4016
7.4213
7.4064
7.3732
7.1762
5.8902
5.3039
4.1987
4.1896
3.7659
3.7544
3.6559
2.0469
1.2728
1.2579
0.0005
JEOL
Filename = sba_110927_1-x
Author = delta
Experiment = single_pulse.
Sample_id = 1-x
Solvent = CHLOROFORM-D
Creation_time = 27-SEP-2011
Revision_time = 6-MAR-2012
Current_time = 6-MAR-2012
Comment = single_pulse
Data_format = 1D COMPLEX
Dim_size = 13107
Dim_title = 1H
Dim_units = [ppm]
Dimensions = X
Site = ECX 500M
Spectrometer = DELTA2_NMR
Field_strength = 11.7473579[T]
X_acq_duration = 1.74587904[s]
X_domain = 1H
X_freq = 500.15991521
X_offset = 7.0[ppm]
X_points = 16384
X_prescans = 1
X_resolution = 0.57277737[Hz]
X_sweep = 9.38438438
Irr_domain = 1H
Irr_freq = 500.15991521
Irr_offset = 5.0[ppm]
Tri_domain = 1H
Tri_freq = 500.15991521
Tri_offset = 5.0[ppm]
Clipped = FALSE
Mod_return = 1
Scans = 10
Total_scans = 10
X_90_width = 15.66[us]
X_acq_time = 1.74587904[s]
X_angle = 45[deg]
X_atn = 3[dB]
X_pulse = 7.83[us]
Irr_mode = Off
Tri_mode = Off
Dante_presat = FALSE
Initial_wait = 1[s]
Recvr_gain = 34
Relaxation_delay = 5[s]
Repetition_time = 6.74587904[s]
Temp_get = 21.1[dC]

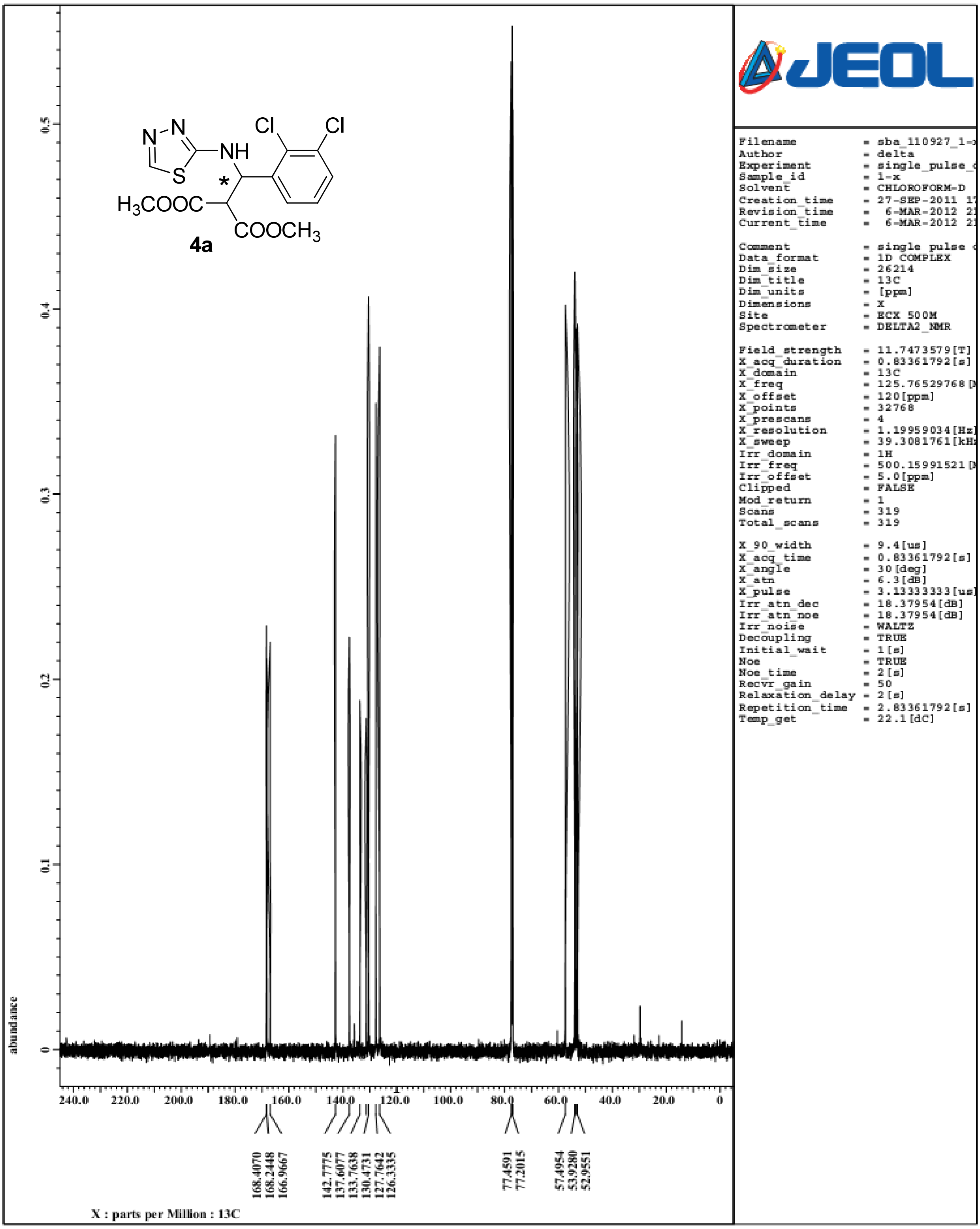
N–N
Cl
Cl
NH
S
*
H3COOC
COOCH3
4a
JEOL
Filename = sba_110927_1-
Author = delta
Experiment = single_pulse_
Sample_id = 1-x
Solvent = CHLOROFORM-D
Creation_time = 27-SEP-2011 1
Revision_time = 6-MAR-2012 2
Current_time = 6-MAR-2012 2
Comment = single pulse
Data_format = 1D COMPLEX
Dim_size = 26214
Dim_title = 13C
Dim_units = [ppm]
Dimensions = X
Site = ECX 500M
Spectrometer = DELTA2_NMR
Field_strength = 11.7473579[T]
X_acq_duration = 0.83361792[s]
X_domain = 13C
X_freq = 125.76529768
X_offset = 120[ppm]
X_points = 32768
X_prescans = 4
X_resolution = 1.19959034[Hz]
X_sweep = 39.3081761[kH
Irr_domain = 1H
Irr_freq = 500.15991521
Irr_offset = 5.0[ppm]
Clipped = FALSE
Mod_return = 1
Scans = 319
Total_scans = 319
X_90_width = 9.4[us]
X_acq_time = 0.83361792[s]
X_angle = 30[deg]
X_atn = 6.3[dB]
X_pulse = 3.13333333[us]
Irr_atn_dec = 18.37954[dB]
Irr_atn_noe = 18.37954[dB]
Irr_noise = WALTZ
Decoupling = TRUE
Initial_wait = 1[s]
Noe = TRUE
Noe_time = 2[s]
Recvr_gain = 50
Relaxation_delay = 2[s]
Repetition_time = 2.83361792[s]
Temp_get = 22.1[dC]
abundance
0
0.1
0.2
0.3
0.4
0.5
240.0
220.0
200.0
180.0
160.0
140.0
120.0
100.0
80.0
60.0
40.0
20.0
0
168.4070
168.2448
166.9667
142.7775
137.6077
133.7638
130.4731
127.7642
126.3335
77.4591
77.2015
57.4954
53.9280
52.9551
X : parts per Million : 13C

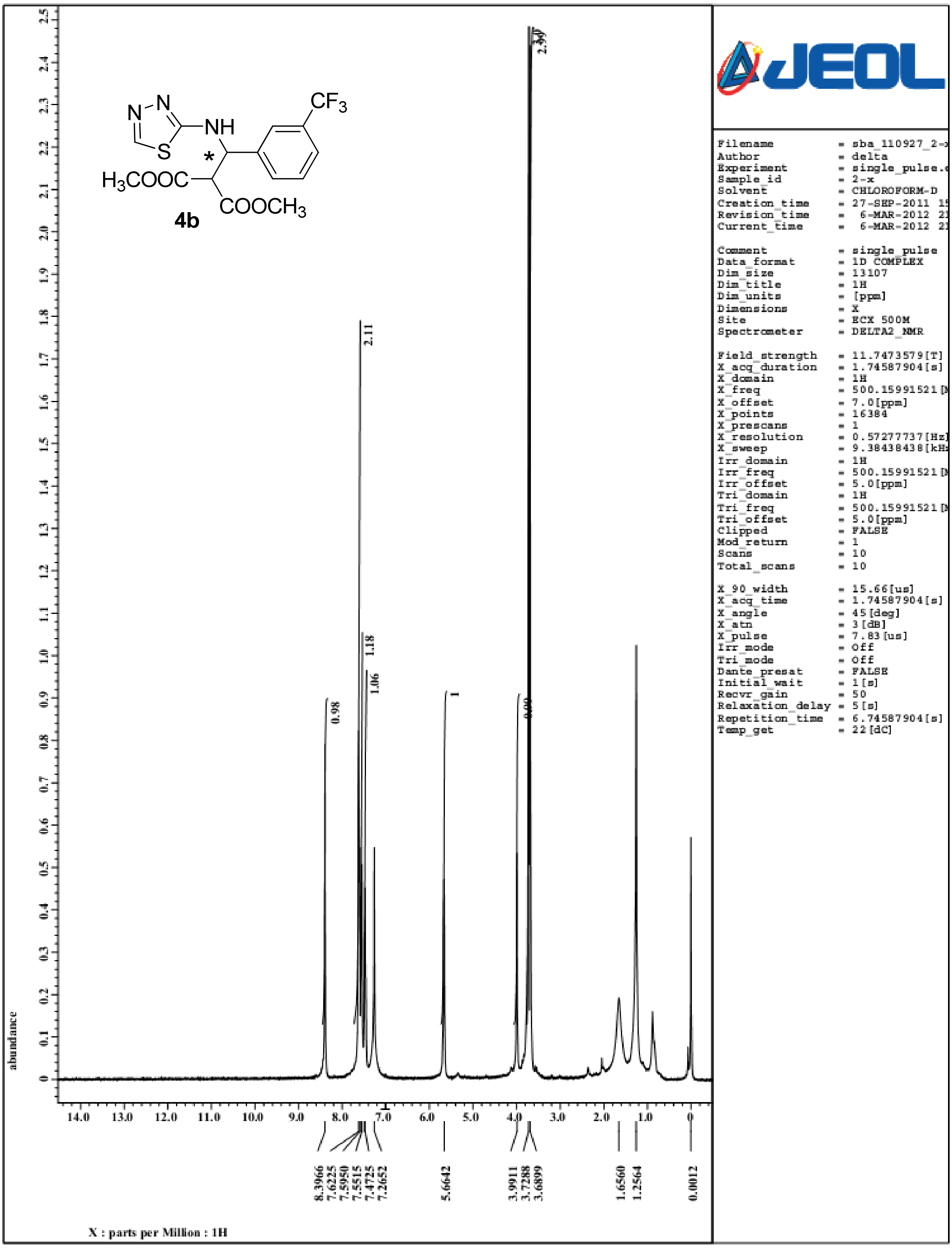

4b
CF3
NH
H3COOC
COOCH3
abundance
X : parts per Million : 1H
8.3966
7.6225
7.5950
7.5515
7.4725
7.2652
5.6642
3.9911
3.7288
3.6899
1.6560
1.2564
0.0012
0.98
2.11
1.18
1.06
1
2.99
JEOL
Filename = sba_110927_2-
Author = delta
Experiment = single_pulse.
Sample_id = 2-x
Solvent = CHLOROFORM-D
Creation_time = 27-SEP-2011
Revision_time = 6-MAR-2012
Current_time = 6-MAR-2012
Comment = single_pulse
Data_format = 1D COMPLEX
Dim_size = 13107
Dim_title = 1H
Dim_units = [ppm]
Dimensions = X
Site = ECX 500M
Spectrometer = DELTA2_NMR
Field_strength = 11.7473579[T]
X_acq_duration = 1.74587904[s]
X_domain = 1H
X_freq = 500.15991521
X_offset = 7.0[ppm]
X_points = 16384
X_prescans = 1
X_resolution = 0.57277737[Hz]
X_sweep = 9.38438438
Irr_domain = 1H
Irr_freq = 500.15991521
Irr_offset = 5.0[ppm]
Tri_domain = 1H
Tri_freq = 500.15991521
Tri_offset = 5.0[ppm]
Clipped = FALSE
Mod_return = 1
Scans = 10
Total_scans = 10
X_90_width = 15.66[us]
X_acq_time = 1.74587904[s]
X_angle = 45[deg]
X_atn = 3[dB]
X_pulse = 7.83[us]
Irr_mode = Off
Tri_mode = Off
Dante_presat = FALSE
Initial_wait = 1[s]
Recvr_gain = 50
Relaxation_delay = 5[s]
Repetition_time = 6.74587904[s]
Temp_get = 22[dC]

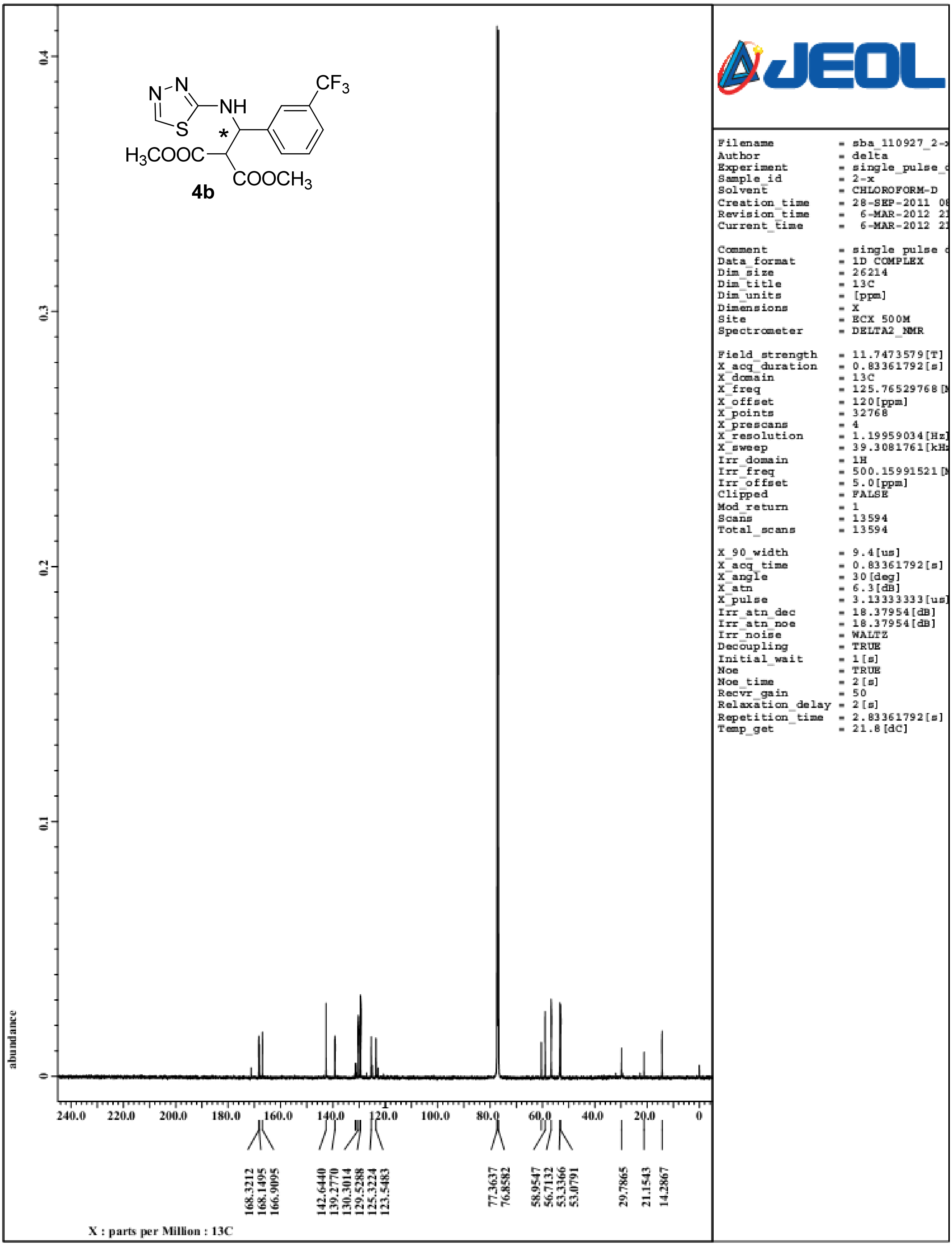
CF3
NH
N
N
S
*
H3COOC
COOCH3
4b
abundance
0.4
0.3
0.2
0.1
0
240.0 220.0 200.0 180.0 160.0 140.0 120.0 100.0 80.0 60.0 40.0 20.0 0
168.3212
168.1495
166.9095
142.6440
139.2770
130.3014
129.5288
125.3224
123.5483
77.3637
76.8582
58.9547
56.7132
53.3366
53.0791
29.7865
21.1543
14.2867
X : parts per Million : 13C
JEOL
Filename = sba_110927_2-
Author = delta
Experiment = single_pulse_
Sample_id = 2-x
Solvent = CHLOROFORM-D
Creation_time = 28-SEP-2011 0
Revision_time = 6-MAR-2012 2
Current_time = 6-MAR-2012 2
Comment = single pulse
Data_format = 1D COMPLEX
Dim_size = 26214
Dim_title = 13C
Dim_units = [ppm]
Dimensions = X
Site = ECX 500M
Spectrometer = DELTA2_NMR
Field_strength = 11.7473579[T]
X_acq_duration = 0.83361792[s]
X_domain = 13C
X_freq = 125.76529768
X_offset = 120[ppm]
X_points = 32768
X_prescans = 4
X_resolution = 1.19959034[Hz]
X_sweep = 39.3081761[kH
Irr_domain = 1H
Irr_freq = 500.15991521
Irr_offset = 5.0[ppm]
Clipped = FALSE
Mod_return = 1
Scans = 13594
Total_scans = 13594
X_90_width = 9.4[us]
X_acq_time = 0.83361792[s]
X_angle = 30[deg]
X_atn = 6.3[dB]
X_pulse = 3.13333333[us]
Irr_atn_dec = 18.37954[dB]
Irr_atn_noe = 18.37954[dB]
Irr_noise = WALTZ
Decoupling = TRUE
Initial_wait = 1[s]
Noe = TRUE
Noe_time = 2[s]
Recvr_gain = 50
Relaxation_delay = 2[s]
Repetition_time = 2.83361792[s]
Temp_get = 21.8[dC]

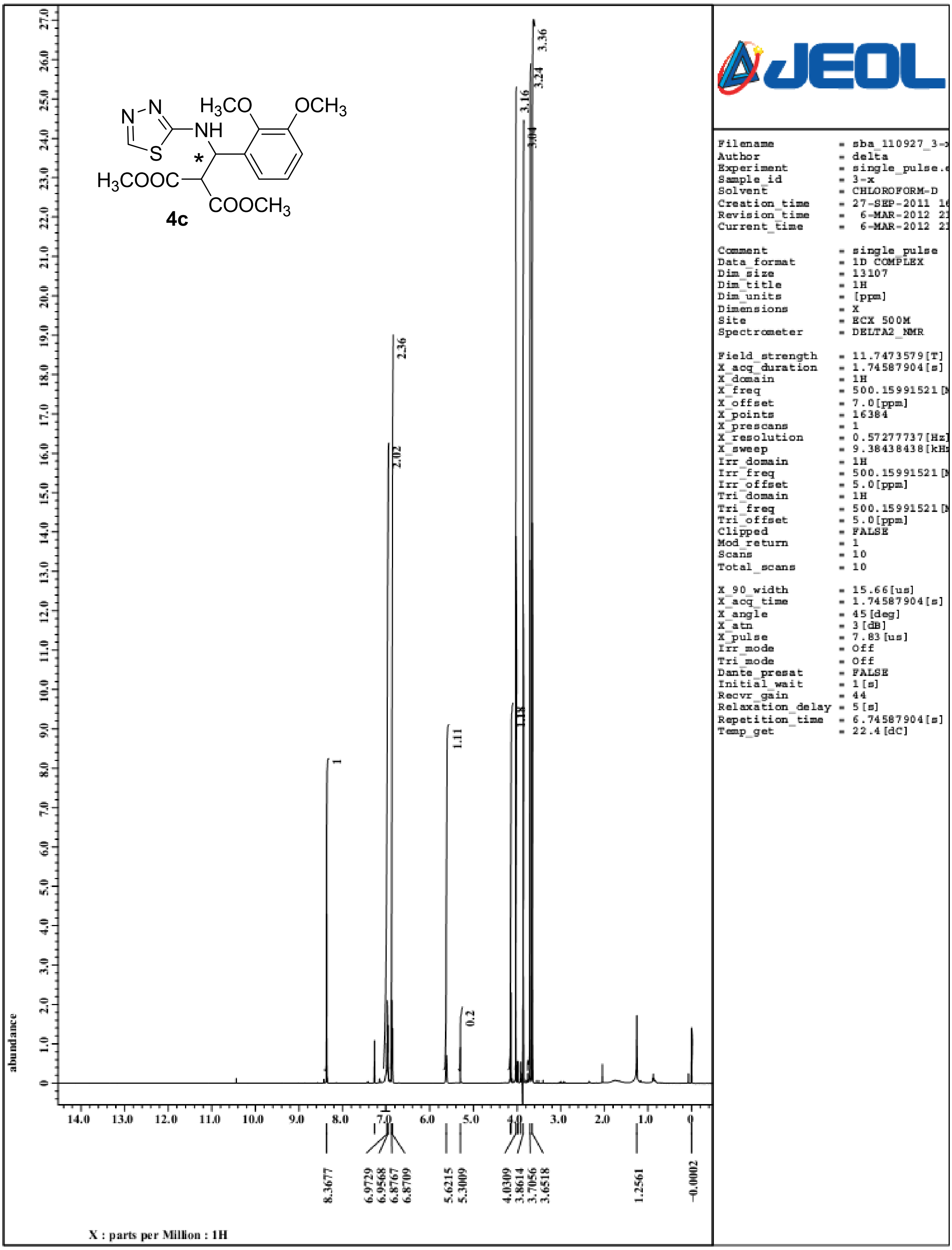
4c
abundance
X : parts per Million : 1H
8.3677
6.9729
6.9568
6.8767
6.8709
5.6215
5.3009
4.0309
3.8614
3.7056
3.6518
1.2561
−0.0002
1
2.02
2.36
1.11
0.2
1.18
3.16
3.04
3.24
3.36
Filename = sba_110927_3-
Author = delta
Experiment = single_pulse.
Sample_id = 3-x
Solvent = CHLOROFORM-D
Creation_time = 27-SEP-2011
Revision_time = 6-MAR-2012
Current_time = 6-MAR-2012
Comment = single_pulse
Data_format = 1D COMPLEX
Dim_size = 13107
Dim_title = 1H
Dim_units = [ppm]
Dimensions = X
Site = ECX 500M
Spectrometer = DELTA2_NMR
Field_strength = 11.7473579[T]
X_acq_duration = 1.74587904[s]
X_domain = 1H
X_freq = 500.15991521
X_offset = 7.0[ppm]
X_points = 16384
X_prescans = 1
X_resolution = 0.57277737[Hz]
X_sweep = 9.38438438
Irr_domain = 1H
Irr_freq = 500.15991521
Irr_offset = 5.0[ppm]
Tri_domain = 1H
Tri_freq = 500.15991521
Tri_offset = 5.0[ppm]
Clipped = FALSE
Mod_return = 1
Scans = 10
Total_scans = 10
X_90_width = 15.66[us]
X_acq_time = 1.74587904[s]
X_angle = 45[deg]
X_atn = 3[dB]
X_pulse = 7.83[us]
Irr_mode = Off
Tri_mode = Off
Dante_presat = FALSE
Initial_wait = 1[s]
Recvr_gain = 44
Relaxation_delay = 5[s]
Repetition_time = 6.74587904[s]
Temp_get = 22.4[dC]

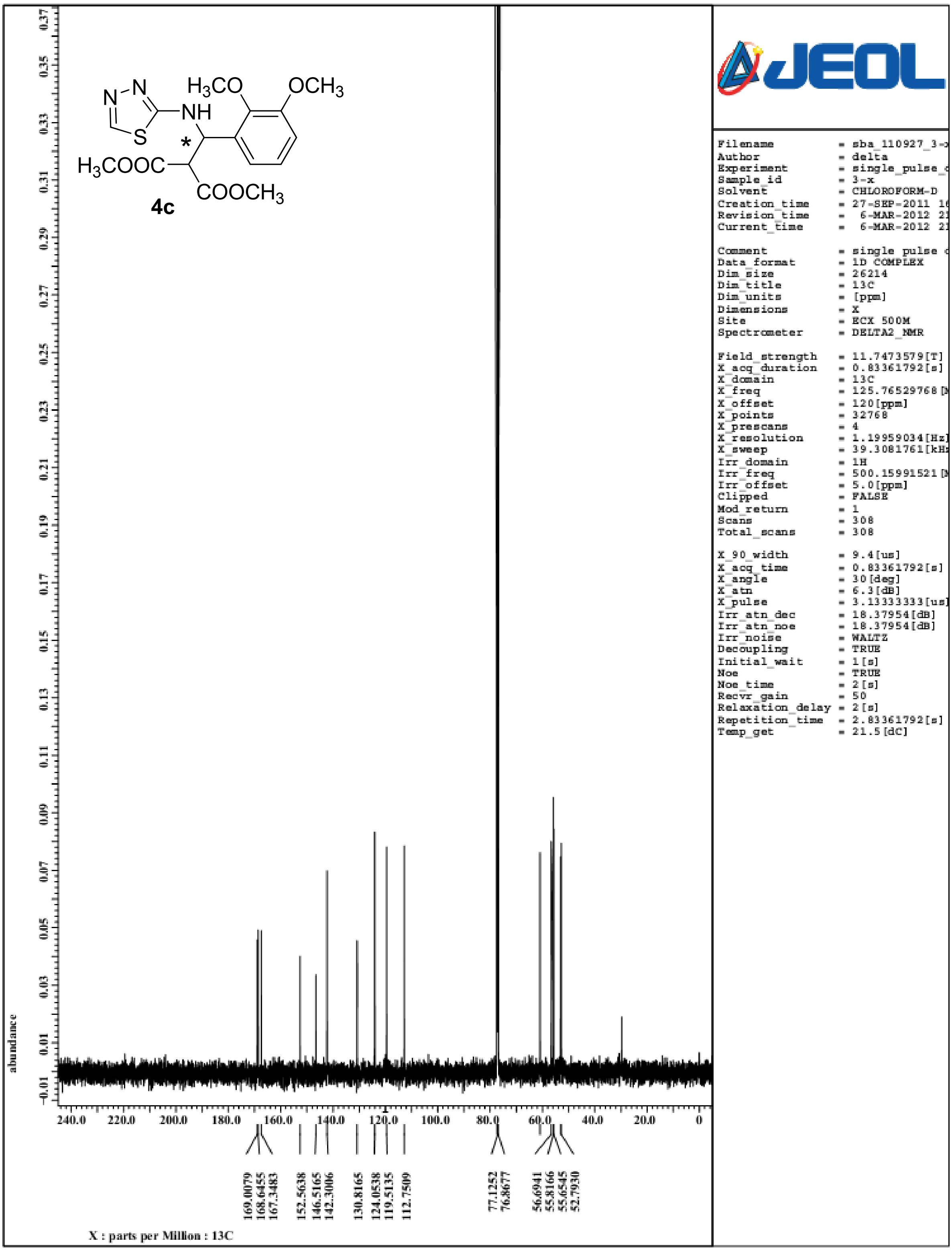
N
N
S
NH
H3CO
OCH3
*
H3COOC
COOCH3
4c
abundance
X : parts per Million : 13C
169.0079
168.6455
167.3483
152.5638
146.5165
142.3006
130.8165
124.0538
119.5135
112.7509
77.1252
76.8677
56.6941
55.8166
55.6545
52.7930
Filename = sba_110927_3-
Author = delta
Experiment = single_pulse_
Sample_id = 3-x
Solvent = CHLOROFORM-D
Creation_time = 27-SEP-2011
Revision_time = 6-MAR-2012
Current_time = 6-MAR-2012
Comment = single pulse
Data_format = 1D COMPLEX
Dim_size = 26214
Dim_title = 13C
Dim_units = [ppm]
Dimensions = X
Site = ECX 500M
Spectrometer = DELTA2_NMR
Field_strength = 11.7473579[T]
X_acq_duration = 0.83361792[s]
X_domain = 13C
X_freq = 125.76529768
X_offset = 120[ppm]
X_points = 32768
X_prescans = 4
X_resolution = 1.19959034[Hz]
X_sweep = 39.3081761
Irr_domain = 1H
Irr_freq = 500.15991521
Irr_offset = 5.0[ppm]
Clipped = FALSE
Mod_return = 1
Scans = 308
Total_scans = 308
X_90_width = 9.4[us]
X_acq_time = 0.83361792[s]
X_angle = 30[deg]
X_atn = 6.3[dB]
X_pulse = 3.13333333[us]
Irr_atn_dec = 18.37954[dB]
Irr_atn_noe = 18.37954[dB]
Irr_noise = WALTZ
Decoupling = TRUE
Initial_wait = 1[s]
Noe = TRUE
Noe_time = 2[s]
Recvr_gain = 50
Relaxation_delay = 2[s]
Repetition_time = 2.83361792[s]
Temp_get = 21.5[dC]

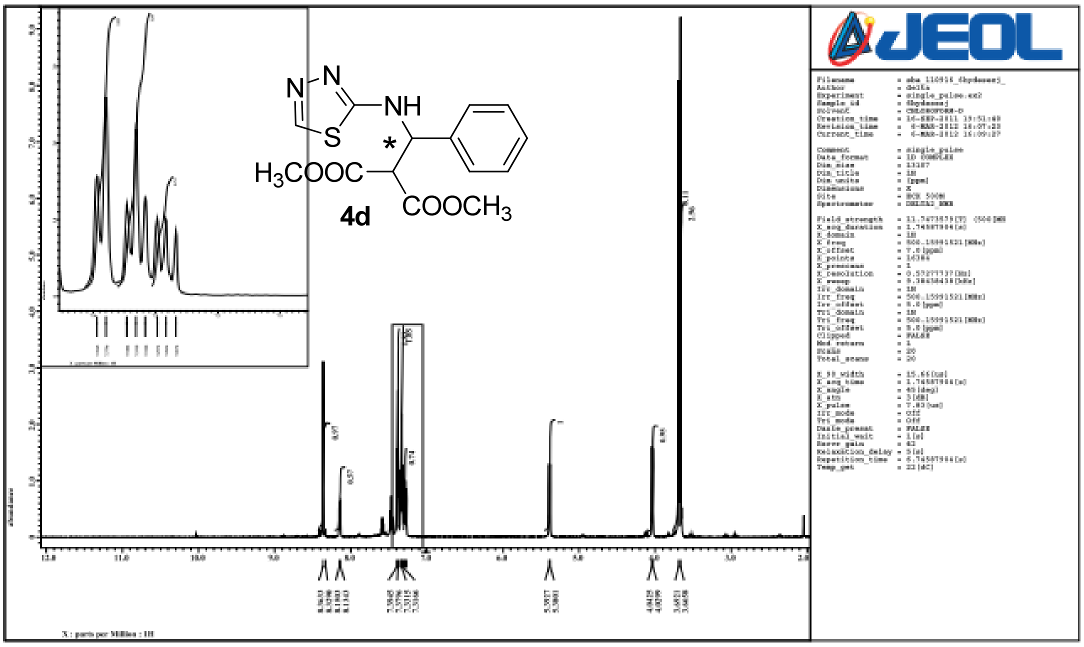

N
N
NH
S
*
H_3COOC
$COOCH_3$
4d
JEOL

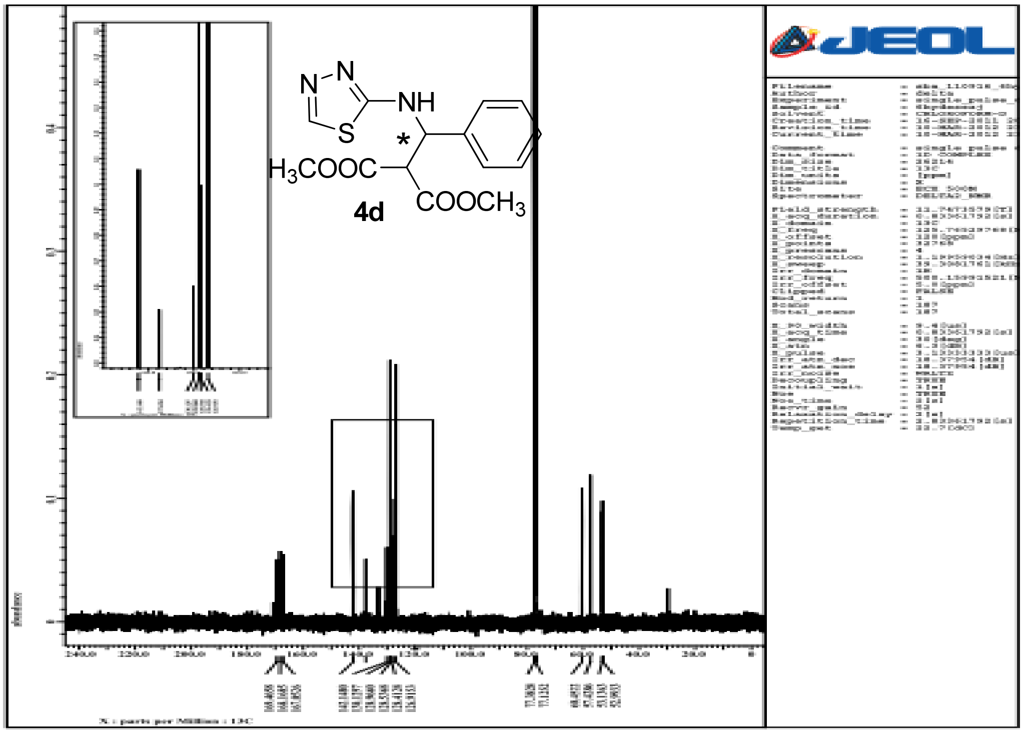

N
N
NH
S
*
H_3COOC
$COOCH_3$
4d
JEOL

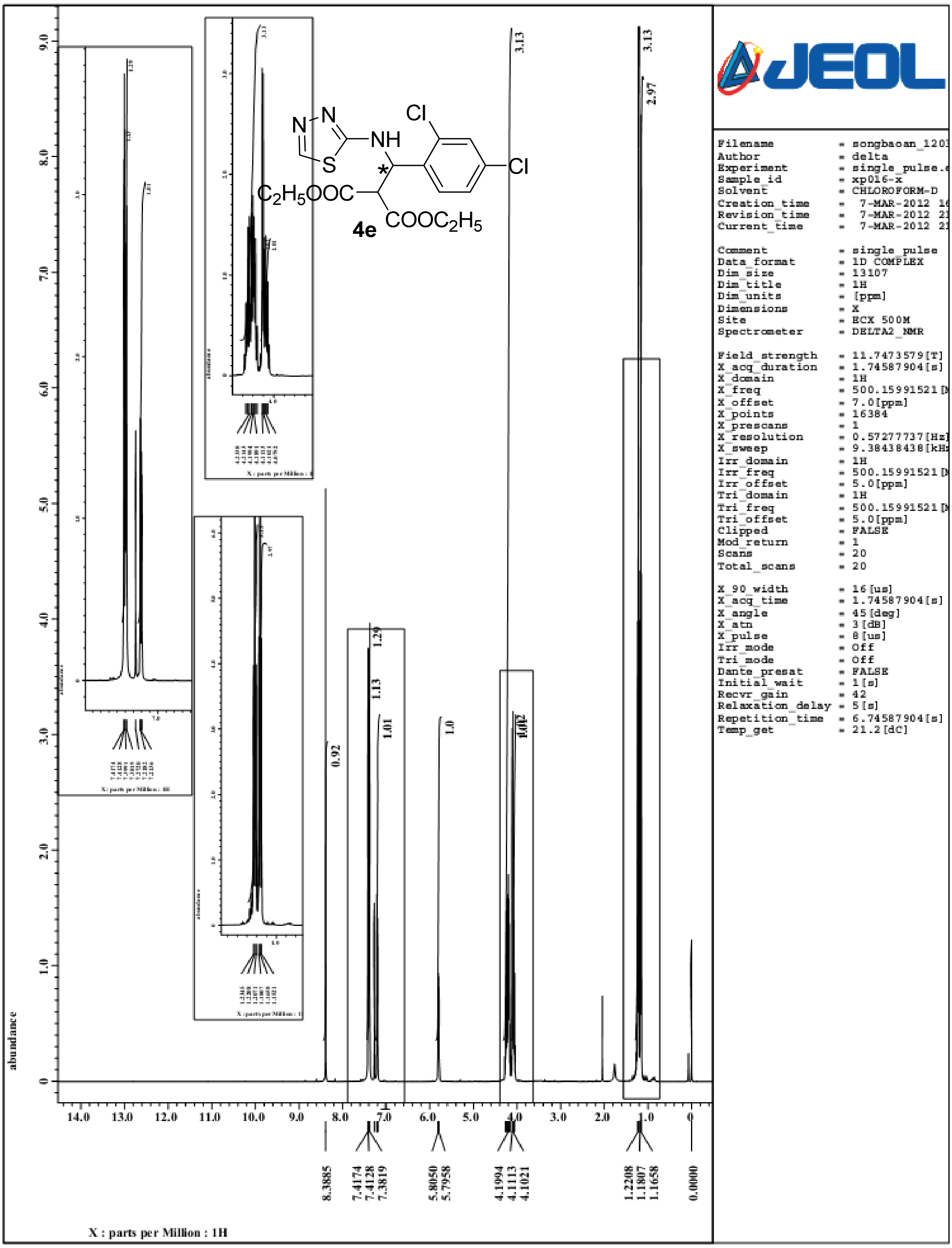
4e
C2H5OOC
COOC2H5
NH
Cl
Filename = songbaoan_120
Author = delta
Experiment = single_pulse.
Sample_id = xp016-x
Solvent = CHLOROFORM-D
Creation_time = 7-MAR-2012
Revision_time = 7-MAR-2012
Current_time = 7-MAR-2012
Comment = single_pulse
Data_format = 1D COMPLEX
Dim_size = 13107
Dim_title = 1H
Dim_units = [ppm]
Dimensions = X
Site = ECX 500M
Spectrometer = DELTA2_NMR
Field_strength = 11.7473579[T]
X_acq_duration = 1.74587904[s]
X_domain = 1H
X_freq = 500.15991521
X_offset = 7.0[ppm]
X_points = 16384
X_prescans = 1
X_resolution = 0.57277737[Hz]
X_sweep = 9.38438438[kHz]
Irr_domain = 1H
Irr_freq = 500.15991521
Irr_offset = 5.0[ppm]
Tri_domain = 1H
Tri_freq = 500.15991521
Tri_offset = 5.0[ppm]
Clipped = FALSE
Mod_return = 1
Scans = 20
Total_scans = 20
X_90_width = 16[us]
X_acq_time = 1.74587904[s]
X_angle = 45[deg]
X_atn = 3[dB]
X_pulse = 8[us]
Irr_mode = Off
Tri_mode = Off
Dante_presat = FALSE
Initial_wait = 1[s]
Recvr_gain = 42
Relaxation_delay = 5[s]
Repetition_time = 6.74587904[s]
Temp_get = 21.2[dC]
abundance
X : parts per Million : 1H
8.3885
7.4174
7.4128
7.3819
5.8050
5.7958
4.1994
4.1113
4.1021
1.2208
1.1807
1.1658
0.0000
0.92
1.13
1.29
1.01
1.0
3.13
2.97

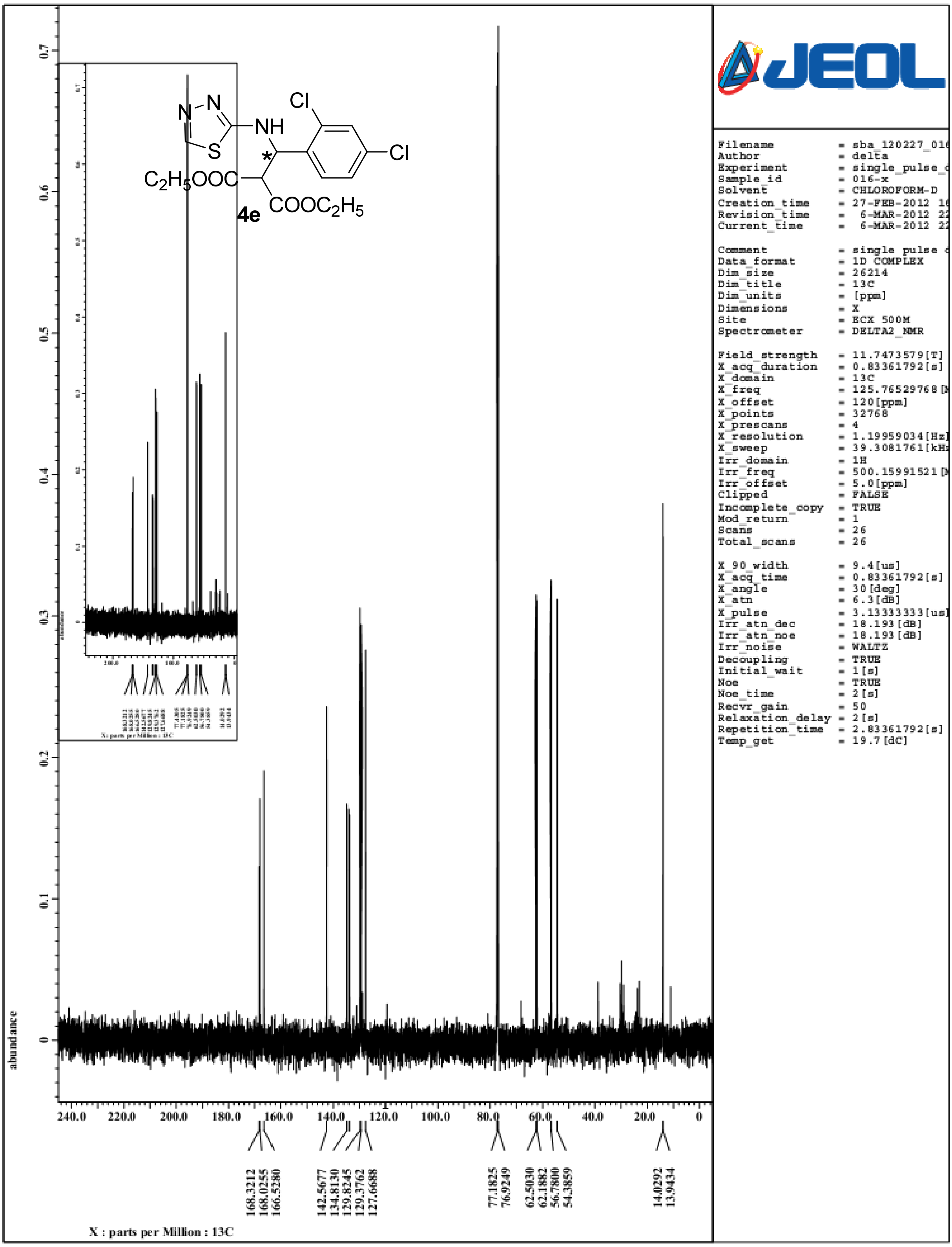

N
N
S
NH
Cl
Cl
*
C_2H_5OOC
$COOC_2H_5$
4e
abundance
X : parts per Million : 13C
168.3212
168.0255
166.5280
142.5677
134.8130
129.8245
129.3762
127.6688
77.1825
76.9249
62.5030
62.1882
56.7800
54.3859
14.0292
13.9434
Filename = sba_120227_016
Author = delta
Experiment = single_pulse_
Sample_id = 016-x
Solvent = CHLOROFORM-D
Creation_time = 27-FEB-2012
Revision_time = 6-MAR-2012
Current_time = 6-MAR-2012
Comment = single pulse
Data_format = 1D COMPLEX
Dim_size = 26214
Dim_title = 13C
Dim_units = [ppm]
Dimensions = X
Site = ECX 500M
Spectrometer = DELTA2_NMR
Field_strength = 11.7473579[T]
X_acq_duration = 0.83361792[s]
X_domain = 13C
X_freq = 125.76529768
X_offset = 120[ppm]
X_points = 32768
X_prescans = 4
X_resolution = 1.19959034[Hz]
X_sweep = 39.3081761[kHz
Irr_domain = 1H
Irr_freq = 500.15991521
Irr_offset = 5.0[ppm]
Clipped = FALSE
Incomplete_copy = TRUE
Mod_return = 1
Scans = 26
Total_scans = 26
X_90_width = 9.4[us]
X_acq_time = 0.83361792[s]
X_angle = 30[deg]
X_atn = 6.3[dB]
X_pulse = 3.13333333[us]
Irr_atn_dec = 18.193[dB]
Irr_atn_noe = 18.193[dB]
Irr_noise = WALTZ
Decoupling = TRUE
Initial_wait = 1[s]
Noe = TRUE
Noe_time = 2[s]
Recvr_gain = 50
Relaxation_delay = 2[s]
Repetition_time = 2.83361792[s]
Temp_get = 19.7[dC]

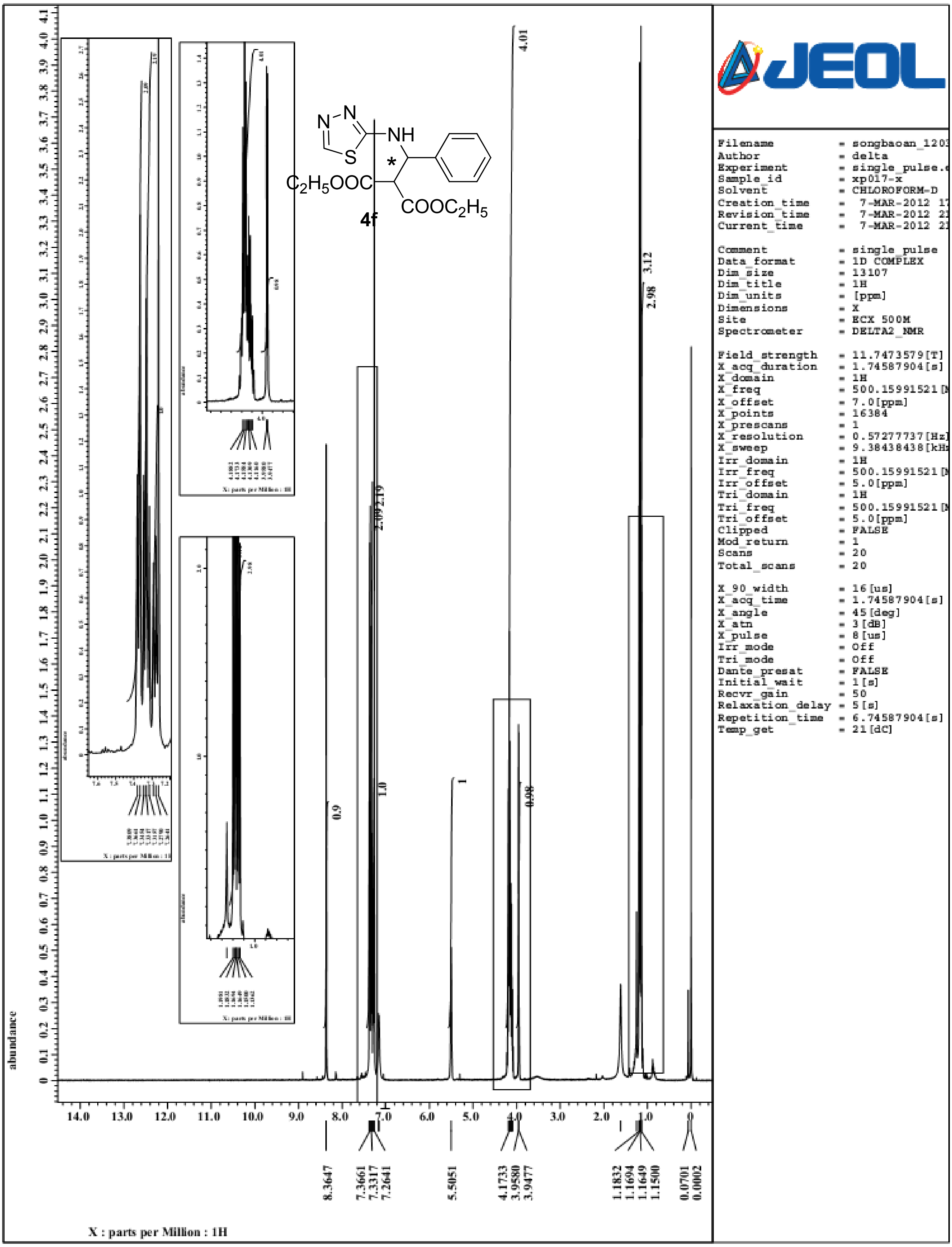

N-N
S
NH
*
C2H5OOC
COOC2H5
4f
abundance
X : parts per Million : 1H
8.3647
7.3661
7.3317
7.2641
5.5051
4.1733
3.9580
3.9477
1.1832
1.1694
1.1649
1.1500
0.0701
0.0002
0.9
2.09
2.19
1.0
1
4.01
0.98
2.98
3.12
Filename = songbaoan_120
Author = delta
Experiment = single_pulse.
Sample_id = xp017-x
Solvent = CHLOROFORM-D
Creation_time = 7-MAR-2012 1
Revision_time = 7-MAR-2012 2
Current_time = 7-MAR-2012 2
Comment = single_pulse
Data_format = 1D COMPLEX
Dim_size = 13107
Dim_title = 1H
Dim_units = [ppm]
Dimensions = X
Site = ECX 500M
Spectrometer = DELTA2_NMR
Field_strength = 11.7473579[T]
X_acq_duration = 1.74587904[s]
X_domain = 1H
X_freq = 500.15991521[M
X_offset = 7.0[ppm]
X_points = 16384
X_prescans = 1
X_resolution = 0.57277737[Hz]
X_sweep = 9.38438438[kHz
Irr_domain = 1H
Irr_freq = 500.15991521[M
Irr_offset = 5.0[ppm]
Tri_domain = 1H
Tri_freq = 500.15991521[M
Tri_offset = 5.0[ppm]
Clipped = FALSE
Mod_return = 1
Scans = 20
Total_scans = 20
X_90_width = 16[us]
X_acq_time = 1.74587904[s]
X_angle = 45[deg]
X_atn = 3[dB]
X_pulse = 8[us]
Irr_mode = Off
Tri_mode = Off
Dante_presat = FALSE
Initial_wait = 1[s]
Recvr_gain = 50
Relaxation_delay = 5[s]
Repetition_time = 6.74587904[s]
Temp_get = 21[dC]

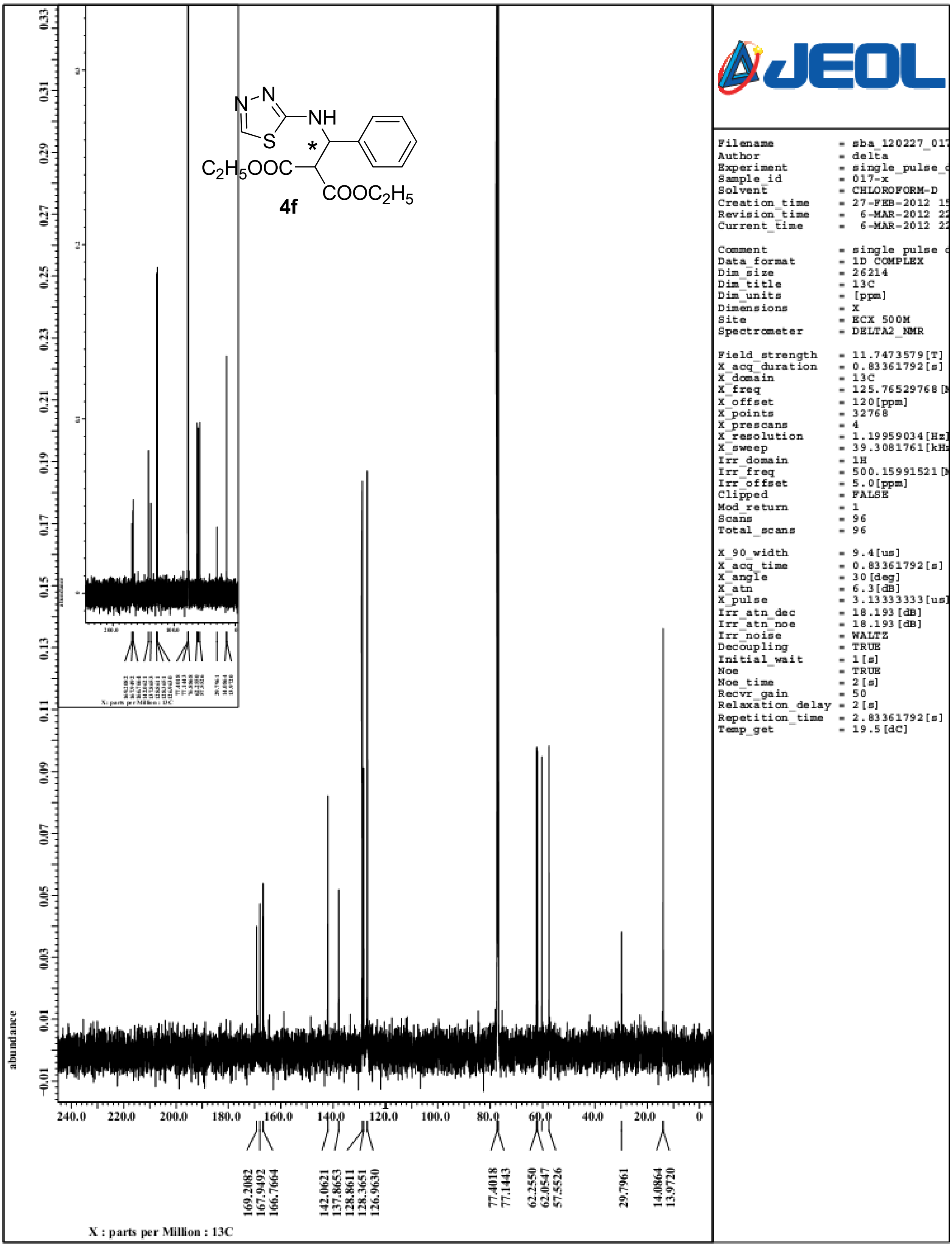
N
N
S
NH
*
C_2H_5OOC
$COOC_2H_5$
4f
JEOL
Filename = sba_120227_017
Author = delta
Experiment = single_pulse_
Sample_id = 017-x
Solvent = CHLOROFORM-D
Creation_time = 27-FEB-2012 15
Revision_time = 6-MAR-2012 22
Current_time = 6-MAR-2012 22
Comment = single pulse
Data_format = 1D COMPLEX
Dim_size = 26214
Dim_title = 13C
Dim_units = [ppm]
Dimensions = X
Site = ECX 500M
Spectrometer = DELTA2_NMR
Field_strength = 11.7473579[T]
X_acq_duration = 0.83361792[s]
X_domain = 13C
X_freq = 125.76529768
X_offset = 120[ppm]
X_points = 32768
X_prescans = 4
X_resolution = 1.19959034[Hz]
X_sweep = 39.3081761[kHz
Irr_domain = 1H
Irr_freq = 500.15991521
Irr_offset = 5.0[ppm]
Clipped = FALSE
Mod_return = 1
Scans = 96
Total_scans = 96
X_90_width = 9.4[us]
X_acq_time = 0.83361792[s]
X_angle = 30[deg]
X_atn = 6.3[dB]
X_pulse = 3.13333333[us]
Irr_atn_dec = 18.193[dB]
Irr_atn_noe = 18.193[dB]
Irr_noise = WALTZ
Decoupling = TRUE
Initial_wait = 1[s]
Noe = TRUE
Noe_time = 2[s]
Recvr_gain = 50
Relaxation_delay = 2[s]
Repetition_time = 2.83361792[s]
Temp_get = 19.5[dC]
abundance
169.2082
167.9492
166.7664
142.0621
137.8653
128.8611
128.3651
126.9630
77.4018
77.1443
62.2550
62.0547
57.5526
29.7961
14.0864
13.9720
X : parts per Million : 13C

2. HPLC Chromatographs of catalyst SQ and 4a–4f

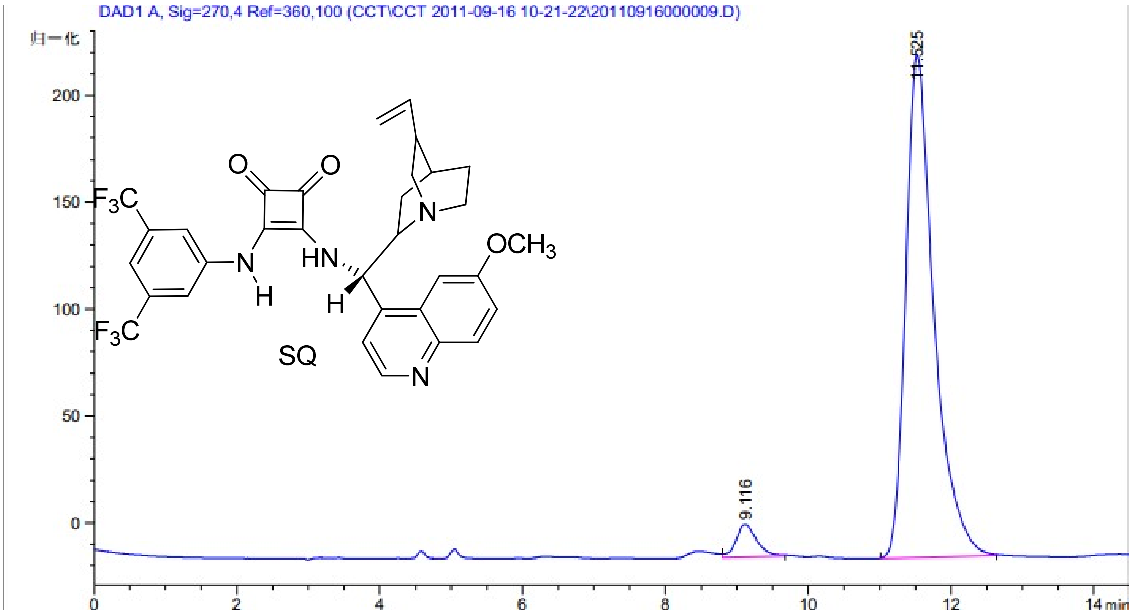

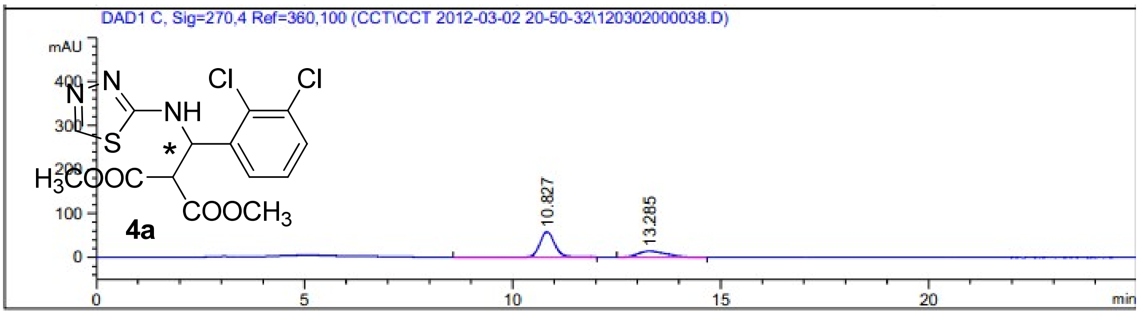

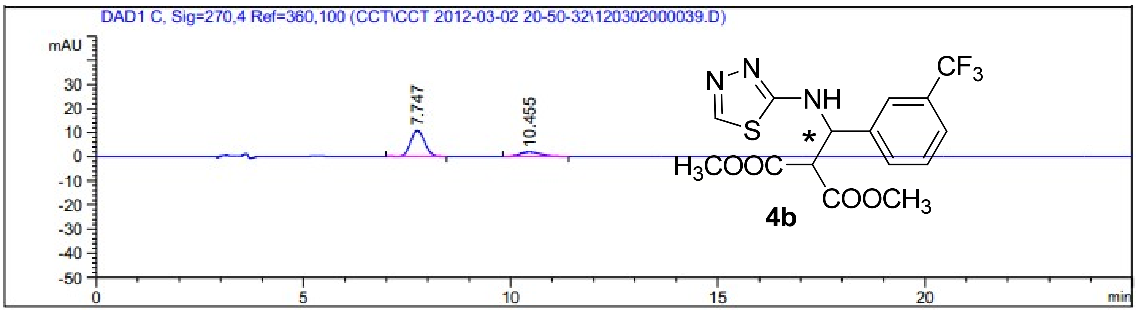

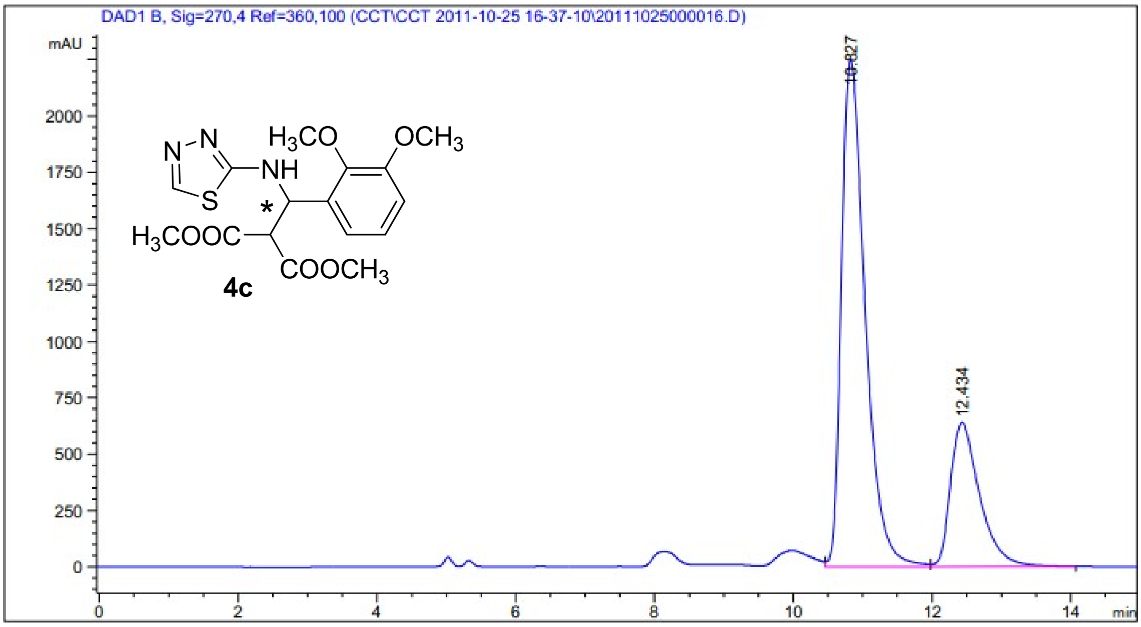
DAD1 B, Sig=270,4 Ref=360,100 (CCT\CCT 2011-10-25 16-37-10\20111025000016.D)
mAU
10.827
12.434
min
4c

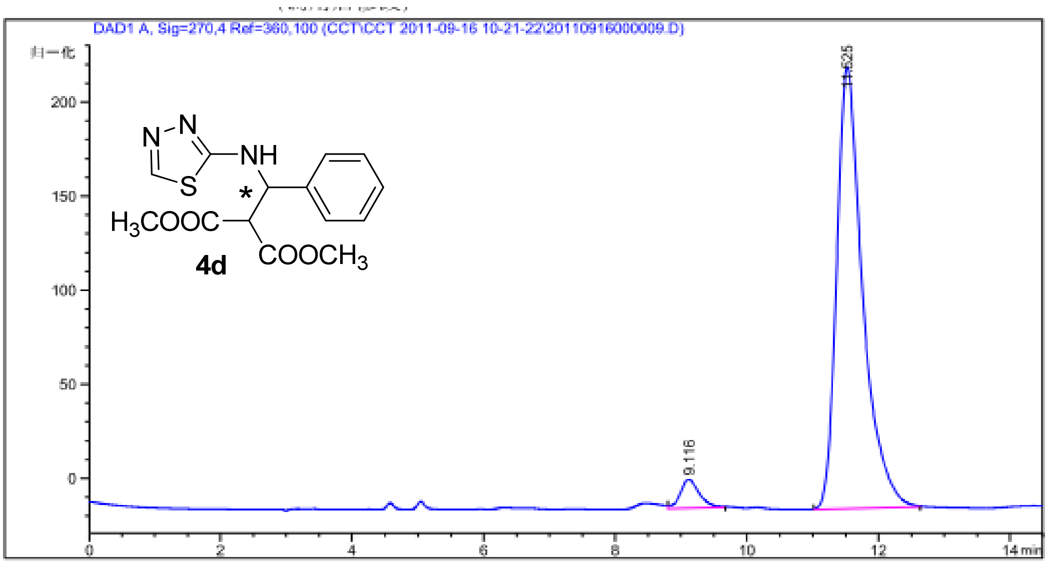
DAD1 A, Sig=270,4 Ref=360,100 (CCT\CCT 2011-09-16 10-21-22\20110916000009.D)
11.525
9.116
14 min
4d

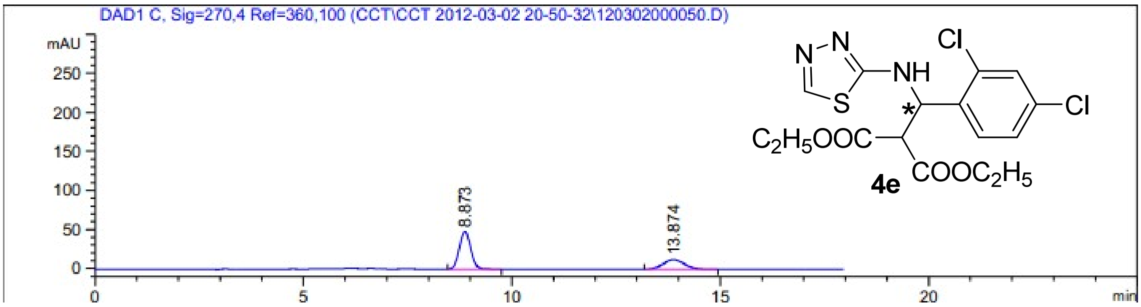
DAD1 C, Sig=270,4 Ref=360,100 (CCT\CCT 2012-03-02 20-50-32\120302000050.D)
mAU
8.873
13.874
min
4e

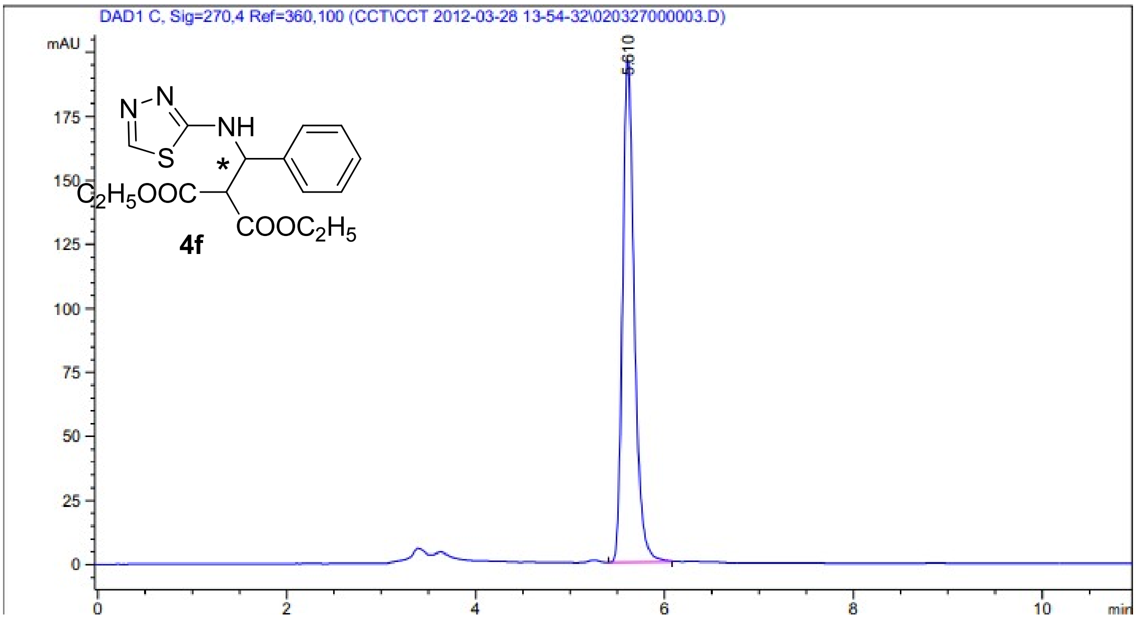
DAD1 C, Sig=270,4 Ref=360,100 (CCT\CCT 2012-03-28 13-54-32\020327000003.D)
mAU
175
150
125
100
75
50
25
0
0
2
4
6
8
10
min
5.610
N
N
NH
S
*
C2H5OOC
COOC2H5
4f
